# Influence of TP53 mutation on efficacy and survival in advanced EGFR‐mutant non‐small cell lung cancer patients treated with third‐generation EGFR tyrosine kinase inhibitors

**DOI:** 10.1002/mco2.586

**Published:** 2024-06-02

**Authors:** Zhonghan Zhang, Jinhui Xue, Yunpeng Yang, Wenfeng Fang, Yan Huang, Shen Zhao, Fan Luo, Jiaxin Cao, Kangmei Zeng, Wenjuan Ma, Jianhua Zhan, Feiteng Lu, Li Zhang, Hongyun Zhao

**Affiliations:** ^1^ Sun Yat‐sen University Cancer Center, State Key Laboratory of Oncology in South China, Guangdong Provincial Clinical Research Center for Cancer Guangzhou China; ^2^ Department of Clinical Research Sun Yat‐sen University Cancer Center State Key Laboratory of Oncology in South China Guangdong Provincial Clinical Research Center for Cancer Guangzhou China; ^3^ Department of Medical Oncology Sun Yat‐sen University Cancer Center State Key Laboratory of Oncology in South China Guangdong Provincial Clinical Research Center for Cancer Guangzhou China; ^4^ Department of Intensive Care Unit Sun Yat‐Sen University Cancer Center State Key Laboratory of Oncology in South China Guangdong Provincial Clinical Research Center for Cancer Guangzhou China; ^5^ Department of Anesthesiology Sun Yat‐Sen University Cancer Center State Key Laboratory of Oncology in South China Guangdong Provincial Clinical Research Center for Cancer Guangzhou China; ^6^ Department of Hematology Oncology and Cancer Immunology Charité ‐ Universitätsmedizin Berlin corporate member of Freie Universität Berlin and Humboldt‐Universität zu Berlin Berlin Germany

**Keywords:** non‐small‐cell lung cancer, targeted therapy, third‐generation EGFR tyrosine kinase inhibitors, TP53 comutation

## Abstract

TP53 comutation is related to poor prognosis of non‐small cell lung cancer. However, there is limited study focusing on the structural influence of TP53 mutation on third‐generation epidermal growth factor receptor tyrosine kinase inhibitors (EGFR‐TKIs) treatment. We retrospectively analyzed the clinical and molecular data of patients treated with third‐generation EGFR‐TKIs in two independent cohorts. A total of 117 patients from the Sun Yat‐sen University Cancer Center (SYSUCC) and 141 patients from the American Association for Cancer Research Project GENIE database were included. In the SYSUCC cohort, TP53 comutations were found in 59 patients (50.4%) and were associated with poor median progress‐free survival (mPFS) and median overall survival (mOS). The additional subtype analysis found that TP53 mutation in the alpha‐helix region had shorter mOS compared with those with TP53 mutations in other regions in the SYSUCC cohort (mOS, 12.2 vs. 21.7 months; *p* = 0.027). Similar findings were confirmed in the GENIE cohort. Specifically, the presence of TP53 mutation in the alpha‐helix region was an independent negative predictive factor for PFS [hazard ratio (HR) 2.05(1.01–4.18), *p* = 0.048] and OS [HR 3.62(1.60–8.17), *p* = 0.002] in the SYSUCC cohort. TP53 mutation in alpha‐helix region was related to inferior clinical outcomes in patients treated with third‐generation EGFR‐TKIs.

## Graphical abstract

TP53 comutation was associated with poor outcomes in non‐small cell lung cancer patients treated with third‐generation epidermal growth factor receptor tyrosine kinase inhibitors. Notably, subgroup analysis has revealed that patients with TP53 mutations in the alpha helix region had shorter median OS compared with those with TP53 mutations in other structural regions. Similar findings were confirmed in another GENIE cohort.

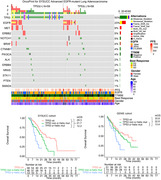

## 1 INTRODUCTION

Epidermal growth factor receptor (EGFR) tyrosine kinase inhibitors (TKIs) are the standard first‐line therapy in advanced EGFR‐mutant non‐small cell lung cancer (NSCLC).^1^, ^2^ Despite high tumor response rates with first‐line EGFR‐TKIs, approximately 60% of patients encounter secondary EGFR T790M mutations at the time of acquired resistance.^3^ Several third‐generation EGFR‐TKIs were designed to selectively and irreversibly target both the original EGFR‐sensitive mutations and the sequential EGFR T790M mutation, such as osimertinib (AZD9291),^4^ aumolertinib (almonertinib; HS‐10296),^5^ avitinib (abivertinib; AC0010),^6^ and so on, which have become the frontline and second‐line therapy for advanced EGFR‐mutant NSCLC. Unfortunately, similar to the situation with first‐generation EGFR‐TKIs, not all the patients reach the average progress‐free survival (PFS) and emerge from a phenomenon of primary drug resistance.^7^ With the widespread use of third‐generation EGFR‐TKIs, elucidation of the molecular mechanisms behind drug resistance and strategies for alleviating it are paramount.

With the development of next‐generation sequencing (NGS), comprehensive genomic analysis could help improve patient outcomes.^2^ TP53 is the most common comutation gene in NSCLC, with frequency ranging from 34.4 to 53.7%.^8^, ^9^ Recently, the TP53 mutation status has been evaluated as a predictor of the clinical outcome of EGFR‐mutated NSCLC patients undergoing treatment with EGFR‐TKIs.^10^, ^11^, ^12^, ^13^, ^14^ Some studies have found an association between TP53 and poorer PFS or overall survival (OS) in the treatment of first‐generation EGFR‐TKIs.^10^, ^11^, ^12^, ^13^, ^14^ Currently, there are also similar findings in some studies on the treatment of third‐generation EGFR‐TKI.^15^, ^16^, ^17^ With the development and wide application of third‐generation TKIs, further data are required to figure out the role of TP53 in the treatment of third‐generation TKIs.

It has been reported that structural TP53 mutations (mut) affect the p53 protein function.^18^, ^19^ However, it is still unclear whether different structural TP53 mutations represent a clinically homogeneous group. Different hotspot TP53 mutants that influence the structure of p53 protein may exert distinct effects on cancer treatment outcomes.^16^, ^20^, ^21^, ^22^ TP53 exon 8 mutation was reported to be related to shorter OS in first‐generation EGFR‐TKIs.^16^, ^20^ However, limited data are available on the effect of TP53 exon 8 in third‐generation EGFR‐TKIs treatment. According to the disturbance of protein structure, TP53 comutation was divided into disruptive and nondisruptive mutations.^21^ Disruptive mutations may lead to loss of function with p53 protein activity, while nondisruptive mutations still retain some of the tumor suppressive functions of p53 protein.^21^ Some studies reported that nondisruptive mutations in TP53 can classify the response of NSCLC receiving first‐line EGFR‐TKIs treatment^11^, ^23^; nevertheless, Molina‐Vila et al.^8^ found the nondisruptive TP53 mutation cannot well predict the prognosis of patients receiving erlotinib. Different classifications of TP53 mutations may have different impacts on prognosis, which warrants further investigation.

Most of the previous studies have primarily focused on examining the variations within the exon regions of TP53 mutation (exon 5−8).^16^, ^20^ Some research has also explored the disruptive/nondisruptive aspects.^8^, ^11^, ^23^ However, there has been a lack of research specifically dedicated to investigating the distribution of secondary structures of the p53 protein. The basic types of protein secondary structure include alpha helix (α‐helix), beta‐strand (β‐strand), loop, turn, and others.^15^, ^24^, ^25^ Alpha‐helix is an important protein secondary structural unit and the formation and stability of the α‐helix are affected by the shape, size, and charge of the side chain groups of the amino acid residues in the peptide chain.^24^ Alpha‐helix is vital for the structure and function of the p53 protein, and structurally changing the α‐helix region might also have some effect on the treatment. The effect of α‐helix alterations due to TP53 mutations on efficacy remains unclear.

This study aimed to confirm the role of the TP53 mutation in advanced NSCLC patients treated with third‐generation EGFR‐TKIs as first‐line treatment or after the failure of a previous EGFR‐TKIs treatment. Analyses were conducted to evaluate the status of the TP53 gene and TP53 subtypes in predicting the prognosis.

## 2 RESULTS

### 2.1 Clinical characteristics of patients and genomic landscape

**FIGURE 1:**
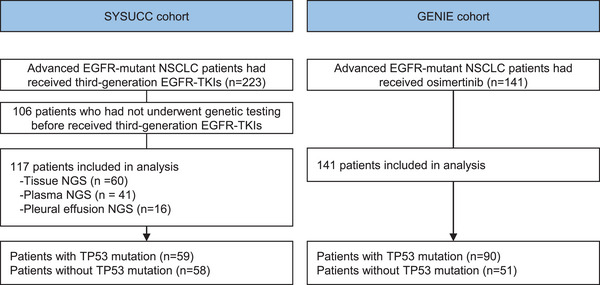
Study flowchart.

Among 223 EGFR‐mutant patients who had received third‐generation EGFR‐TKIs, qualified sequencing data were acquired from 117 patients (Figure 1). A total of 60 patients were genotyped by tissue NGS, 41 by plasma‐based NGS, and 16 by pleural effusion NGS. Table 1 details the clinicopathological characteristics of the patients in the Sun Yat‐sen University Cancer Center (SYSUCC) cohort. The majority of patients were female (62.4%), and 84 were never smokers (71.8%) (Table 1). There were 107 (91.5%) patients received osimertinib, and 10 (8.5%) received avitinib or almonertinib. Seventy‐five (64.1%) of EGFR exon 19 deletions (19 del) and 39 (33.3%) of EGFR exon 21 L858R point mutations were detected. A similar proportion of patients were present in the TP53 mut and wild‐type (wt) group based on age, gender, histology, smoking history, EGFR mutations, type of third‐generation TKIs, treatment lines, bone metastases, and brain metastases. Patients in the TP53 mut group had more liver metastases than patients in the TP53 wt group (22.0% vs. 6.9%, *p* = 0.039). The emergence of EGFR T790M mutation was observed in 85 patients; no difference was observed between TP53 wt (72.4%) and TP53 mut (72.9%) groups.

**TABLE 1:** Characteristics of patients with and without TP53 mutations.

No. of patients (%)	Parameter	Overall	TP53 wt	TP53 mut in other region	TP53 mut in α‐helix region	*p* Value
Total		117	58	46	13	
Age (median [IQR])		54.0 [47.0, 64.0]	57.5 [47.5, 66.8]	52.0 [46.2, 61.8]	54.0 [49.0, 58.0]	0.135
Gender (%)	Female	73 (62.4)	38 (65.5)	25 (54.3)	10 (76.9)	0.262
	Male	44 (37.6)	20 (34.5)	21 (45.7)	3 (23.1)	
Histology (%)	Adenocarcinoma	114 (97.4)	57 (98.3)	44 (95.7)	13 (100.0)	0.579
	Squamous	3 (2.6)	1 (1.7)	2 (4.3)	0 (0.0)	
Smoke (%)	No	84 (71.8)	45 (77.6)	29 (63.0)	10 (76.9)	0.238
	Yes	33 (28.2)	13 (22.4)	17 (37.0)	3 (23.1)	
EGFR (%)	EGFR exon 19	75 (64.1)	36 (62.1)	32 (69.6)	7 (53.8)	0.732
	EGFR exon 20	3 (2.6)	2 (3.4)	1 (2.2)	0 (0.0)	
	EGFR exon 21	39 (33.3)	20 (34.5)	13 (28.3)	6 (46.2)	
T790M (%)	No	32 (27.4)	16 (27.6)	15 (32.6)	1 (7.7)	0.205
	Yes	85 (72.6)	42 (72.4)	31 (67.4)	12 (92.3)	
TKIs (%)	Osimertinib	107 (91.5)	50 (86.2)	44 (95.7)	13 (100.0)	0.117
	Other	10 (8.5)	8 (13.8)	2 (4.3)	0 (0.0)	
Treatment lines (%)	First‐line	9 (7.7)	6 (10.3)	3 (6.5)	0 (0.0)	0.626
	Second‐line	70 (59.8)	36 (62.1)	26 (56.5)	8 (61.5)	
	≥Third‐line	38 (32.5)	16 (27.6)	17 (37.0)	5 (38.5)	
Metastatic sites						
Brain (%)	No	69 (59.0)	34 (58.6)	28 (60.9)	7 (53.8)	0.899
	Yes	48 (41.0)	24 (41.4)	18 (39.1)	6 (46.2)	
Liver (%)	No	100 (85.5)	54 (93.1)	38 (82.6)	8 (61.5)	0.039^a^
	Yes	17 (14.5)	4 (6.9)	8 (17.4)	5 (38.5)	
Bone (%)	No	58 (49.6)	30 (51.7)	24 (52.2)	4 (30.8)	0.355
	Yes	59 (50.4)	28 (48.3)	22 (47.8)	9 (69.2)	
Pleural effusion (%)	No	102 (87.2)	50 (86.2)	42 (91.3)	10 (76.9)	0.373
	Yes	15 (12.8)	8 (13.8)	4 (8.7)	3 (23.1)	

Abbreviations: IQR, interquartile range; TKIs, tyrosine kinase inhibitors; TP53 mut, TP53 mutation; TP53 wt, TP53 wild‐type.

^a^ Chi‐square tests for TP53mut versus TP53wt cases.

**FIGURE 2:**
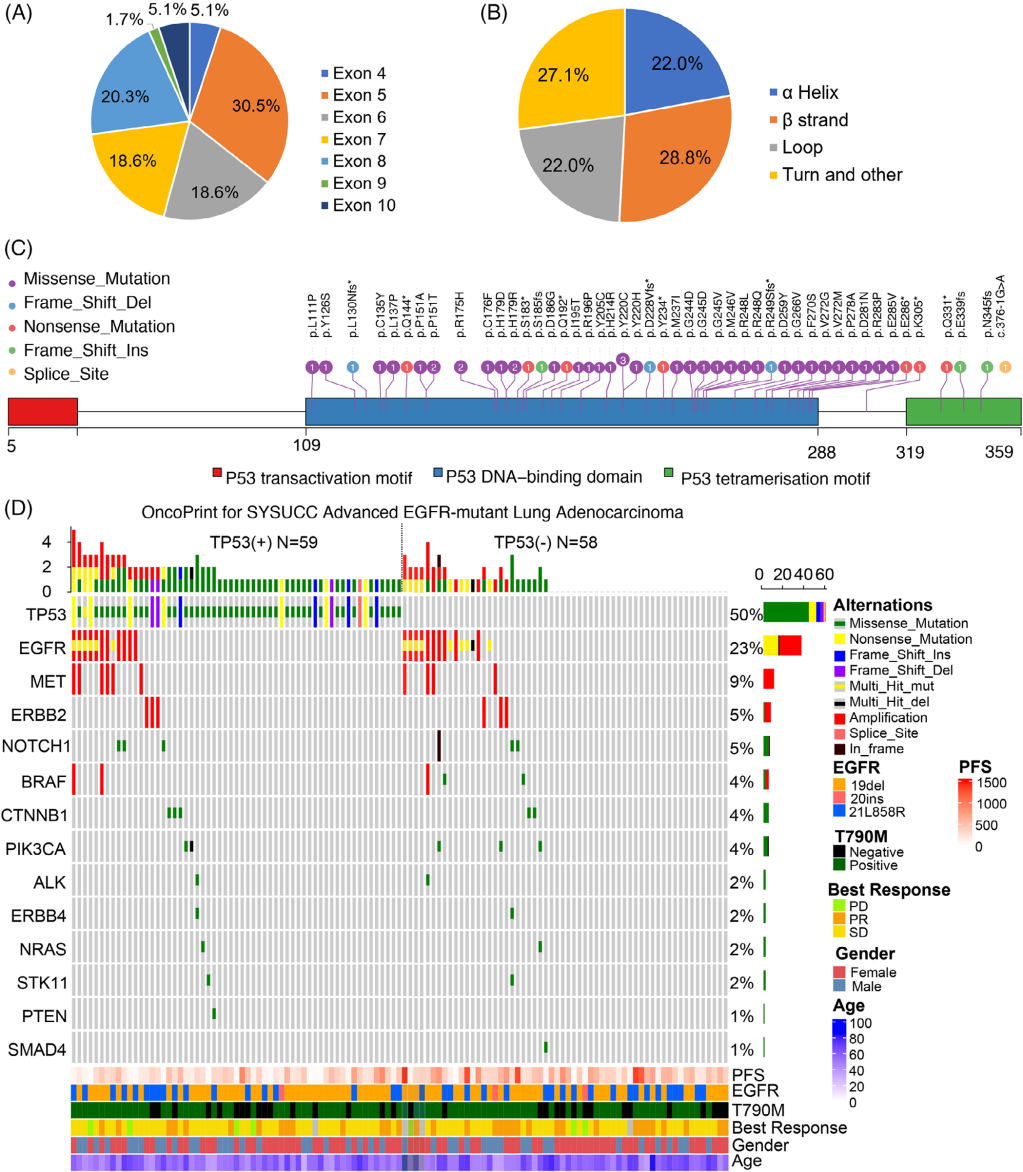
Genomic profiling of TP53 mutations in advanced NSCLC treated with third‐generation EGFR‐TKIs. (A) Pie chart showing the Exons and (B) the structure region distribution of TP53 mutations. (C) Lollipop plot demonstrating all TP53 variants in this cohort (*n* = 59). The number of times the variant observed in this current cohort was displayed within the lollipops. (D) Oncoprint heatmap illustrating 117 EGFR‐mutant lung adenocarcinoma carcinoma from this dataset. Only a subset of genes are shown from the MyGene 22 lung cancer‐related genes panel.

Of the 59 patients with TP53 mutation detected, the mutation in TP53 exon 5 (30.5%) was the most common, followed by exon 8 (20.3%), exon 7 (18.6%), exon 6 (18.6%), and others (Figure 2A). As for the perspective of the structural subgroup, TP53 mutations were classified according to the secondary structure of the TP53 protein, including mutation in the α‐helix, β‐strand, loop, turn and other region. The mutation of the TP53 α‐helix region was present in 22.0% of patients, TP53 β‐strand region in 28.8% of patients, TP53 loop region in 22.0% of patients, turn and other regions in 27.1% of patients (Figure 2B and Table S1). Details of TP53 mutations are shown in Figure 2C. Other coexisting mutations were present in 55 cases, including EGFR amplification (17.9%, 21 out of 117), MET amplification (8.5%, 10 out of 117), NOTCH mutation/In frame del (5.1%, six out of 117), and BRAF amplification/mutation(4.3%, five out of 117), as shown in Figure 2D.

In the independent cohort from the American Association for Cancer Research Project GENIE database, a total of 141 patients with EGFR‐mutant NSCLC who received osimertinib were included in this study (Table S2). There were no differences in baseline characteristics between TP53 mut and TP53 wt patients in this cohort.

### 2.2 TP53 mutation was related to shorter median PFS and mOS of NSCLC patients treated with third‐generation EGFR‐TKIs

**FIGURE 3:**
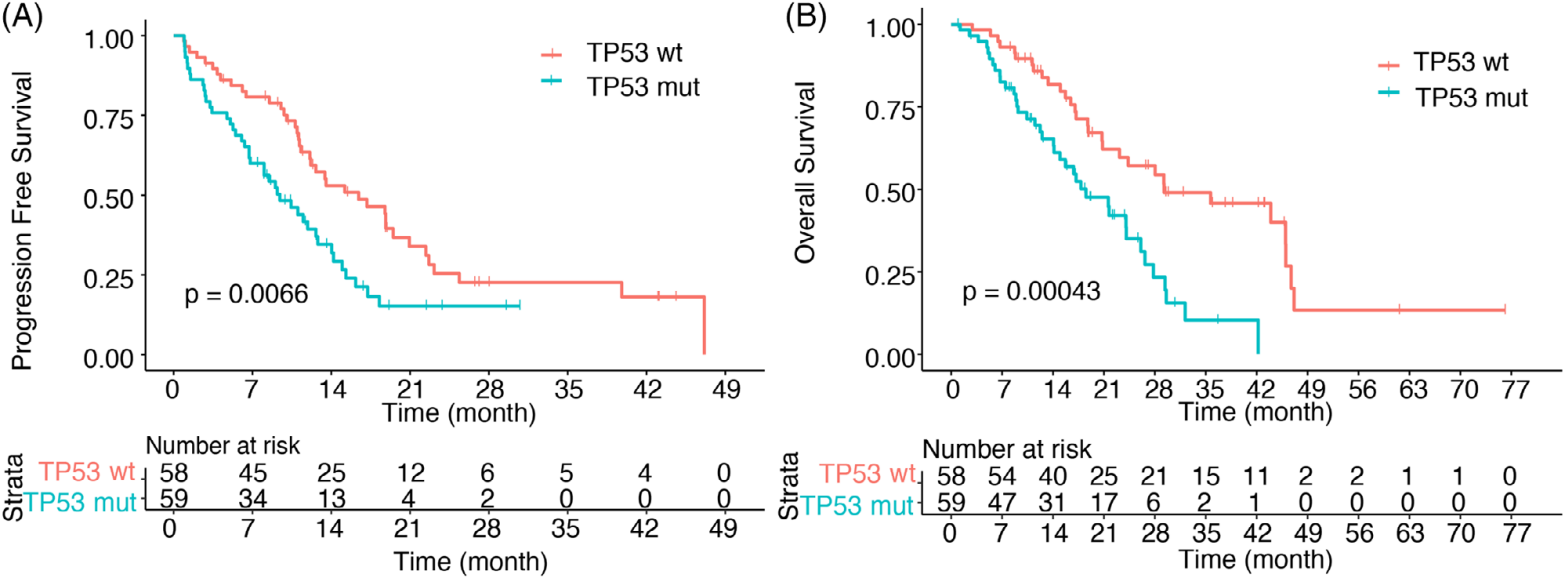
Kaplan–Meier curves of progression‐free survival and overall survival of advanced NSCLC treated with third‐generation EGFR‐TKIs based on TP53 mutation status. (A) Kaplan–Meier survival analysis of progression‐free survival according to the state of TP53 mutations. (B) Kaplan–Meier survival analysis of overall survival according to the state of TP53 mutations. TP53 wt, TP53 wild‐type; TP53 mut, TP53 mutation.

In the SYSUCC cohort, 81 patients reached the PFS event; the median PFS (mPFS) of all patients was 12.2 months [95% confidence intervals (CI), 11.0–15.3]. Patients with TP53 comutation had worse mPFS than TP53 wt patients (mPFS, 9.4 vs. 16.4 months, *p* = 0.0066) (Figure 3A). A total of 68 patients reached the OS event; median OS was 24.0 months (95% CI, 18.8–29.3). The presence of TP53 comutation was related to shorter OS (mOS, 18.5 vs. 29.3 months; *p* = 0.00043) (Figure 3B).

### 2.3 TP53 mutation in the alpha‐helix region was associated with inferior clinical outcome

Considering that different hotspot p53 mutants that influence the structure of wt p53 may exert distinct effects on cancer treatment, we further explored the impact on the prognosis of different TP53 subgroups. TP53 exon 8 mut had a relatively worse PFS compared with mutations in other exons (mPFS, 7.4 vs. 10.4 months, *p* = 0.049; Figure S1A), while no significant difference was observed in OS(mOS, 14.8 vs. 21.7 months; *p* = 0.15; Figure S1B). When classifying TP53 mutation according to the disruptive/nondisruptive alterations, the PFS (8.5 vs. 11.5 months; *p* = 0.50) (Figure S1C) and OS (mOS, 17.8 vs. 18.5 months; *p* = 0.86) (Figure S1D) of patients with TP53 nondisruptive mut and disruptive mut had no difference.

**FIGURE 4:**
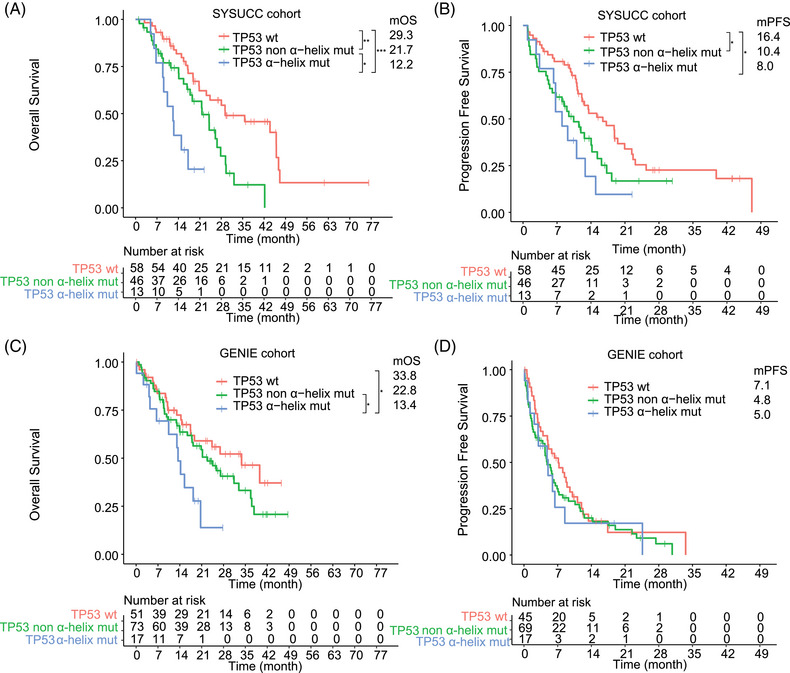
Kaplan–Meier curves of progression‐free survival and overall survival of advanced NSCLC treated with third‐generation EGFR‐TKIs based on structural subgroup  of TP53 mutation. (A) Kaplan–Meier survival analysis of overall survival according to TP53 α‐helix or non‐α‐helix mutation in the Sun Yat‐sen University Cancer Center (SYSUCC) cohort. (B) Kaplan–Meier survival analysis of progression‐free survival according to TP53 α‐helix or non‐α‐helix mutation in SYSUCC cohort. (C) Kaplan–Meier survival analysis of overall survival according to TP53 α‐helix or non‐α‐helix in GENIE cohort. (D) Kaplan–Meier survival analysis of progression‐free survival according to TP53 α‐helix or non‐α‐helix mutation in GENIE cohort. TP53 wt, TP53 wild‐type; TP53 mut, TP53 mutation.

Then, we first paid attention to the influence of the mutations that affect the secondary structure of TP53 protein; the mOS of patients with TP53 mutation in α‐helix region, TP53 β‐strand region, TP53 loops region, TP53 turn and other regions were 12.2, 26.6, 15.7, and 21.6 months, respectively (Figure S2). Notably, we observed that the mOS of patients with TP53 mut in α‐helix region was significantly shorter compared with other TP53 mutations (mOS, 12.2 vs. 21.7 months; *p* = 0.027) or TP53wt cases (mOS, 12.2 vs. 29.3 months; *p* = 0.0006) (Figure 4A). Patients with TP53 mutation in α‐helix region had significantly worse PFS compared with TP53wt cases (mPFS, 8.0 vs. 16.4 months, *p* = 0.021) (Figure 4B). Using a Cox regression analysis, the univariate analysis indicated that TP53 mutation in α‐helix region, TP53 mutation in other regions, and liver metastases were related to shorter PFS (Table 2). After multivariate analysis for the endpoint PFS, TP53 mutation in α‐helix region [hazard ratio (HR), 2.05 (1.01–4.18), *p* = 0.048] and TP53 mutation in other regions [1.67 (1.02–2.71), *p* = 0.04] contributed significantly to the Cox model. Univariate analysis indicated that TP53 mutation in α‐helix region, TP53 mutation in other regions, and liver metastases were associated with inferior OS (Table 2). Using multivariable regression analysis, both TP53 mutation in α‐helix region [HR 3.62 (1.6–8.17), *p* = 0.002], TP53 mutation in other regions [1.87 (1.06–3.31), *p* = 0.03] and liver metastases [HR 1.91 (1.00–3.64), *p* = 0.049] remained significant independent predictors of OS.

**TABLE 2:** Univariate and multivariate Cox analysis of progression‐free survival and overall survival (*n* = 117).

	PFS				OS			
	Univariate		Multivariate		Univariate		Multivariate	
Characteristics	HR (95% CI)	*p* Value	HR (95% CI)	*p* Value	HR (95% CI)	*p* Value	HR (95% CI)	*p* Value
Age	0.99 (0.97–1.01)	0.355			0.99 (0.97–1.01)	0.238		
Gender		0.672				0.483		
Male	1.1 (0.7–1.74)				0.83 (0.50–1.39)			
Female	1(Reference)				1			
Histology		0.927				0.746		
Adenocarcinoma	0.936 (0.229–3.82)				1.39 (0.19–10)			
Squamous cell carcinoma	1(Reference)				1(Reference)			
Smoking		0.723				0.811		
No	1(Reference)				1(Reference)			
Yes	1.09 (0.66–1.8)				1.07 (0.62–1.84)			
EGFR 19DEL		0.255				0.056		
No	1(Reference)				1(Reference)			
Yes	0.77 (0.49–1.21)				0.62 (0.37–1.01)			
EGFR L858R		0.525				0.115		
No	1(Reference)				1(Reference)			
Yes	1.16 (0.73–1.84)				1.5 (0.91–2.49)			
EGFR T790M		0.385				0.925		
No	1(Reference)				1(Reference)			
Yes	0.80 (0.49–1.32)				0.97 (0.57–1.68)			
TP53 status								
TP53 wt	1(Reference)		1(Reference)		1(Reference)		1(Reference)	
TP53 mut in α‐helix region	2.39 (1.21–4.75)	0.013	2.05 (1.01–4.18)	0.048	4.45 (2.05–9.66)	0.0002	3.62 (1.60–8.17)	0.002
TP53 mut in other region	1.72 (1.06–2.79)	0.028	1.67 (1.02–2.71)	0.04	2.06 (1.18–3.58)	0.011	1.87 (1.06–3.31)	0.03
Third‐generation TKI		0.383				0.202		
Osimertinib	1.5 (0.60–3.71)				1.8 (0.73–4.45)			
Avitinib or almonertinib	1(Reference)				1(Reference)			
Metastatic sites								
Liver	2.05 (1.16–3.6)	0.013	1.77 (0.98–3.18)	0.058	2.65 (1.43–4.88)	0.002	1.91 (1.00–3.64)	0.049
Brain	1.24 (0.79–1.93)	0.349			1.52 (0.93–2.49)	0.096		
Bone	1.08 (0.69–1.67)	0.746			1.33 (0.82–2.16)	0.243		
Pleural effusion	1.85 (1.00–3.45)	0.051			1.92 (0.99–3.72)	0.054		

Abbreviations: CI, confidence interval; HR, hazard ratio; OS, overall survival; PFS, progression‐free survival; TP53 mut, TP53 mutation; TP53 wt, TP53 wild‐type.

We also divided the GENIE cohort into patients with TP53 mutation in α‐helix region, TP53 mutation in other region, and TP53 wt group. For OS analyses, data from 141 patients were available. Consistent with the SYSUCC cohort, patients with TP53 mutation in α‐helix region had significantly shorter OS compared with TP53 mutation in other region (mOS, 13.4 vs. 22.8 months, *p* = 0.049) or TP53 wt patients (mOS, 13.4 vs. 33.8 months, *p* = 0.039) (Figure 4C). OS for patients with TP53 mutation in other regions and TP53wt was not different (*p* = 0.21). For PFS analyses, data from 131 patients were available. No difference in mPFS was observed between patients with TP53 mutation in α‐helix region versus TP53 mutation in other region (mPFS, 5.0 vs. 4.8 months, *p* = 0.85) or TP53wt patients (mPFS, 5.0 vs. 7.1 months, *p* = 0.52) (Figure 4D).

### 2.4 Poor mOS was observed in patients with TP53 mut plus EGFR exon 21 L858R mutation

To figure out whether different EGFR mutations affected clinical outcomes, subgroup analyses were conducted in patients with EGFR T790M mutation, EGFR exon 19 deletion, and EGFR 21 L858R mutation. The mPFS of patients with TP53 wt plus T790M mut, TP53 wt plus no T790M mut, TP53 mut plus T790M mut, and TP53 mut plus no T790M mut were 13.4, 18.8, 9.4, and 9.0 months, respectively (Figure S3A). The mOS of patients with TP53 wt plus T790M mut, TP53 wt plus no T790M mut, TP53 mut plus T790M mut, and TP53 mut plus no T790M mut were 29.3, 35.7, 18.5, and 17.8 months, respectively (Figure S3B). Patients in the TP53 wt subgroup appeared to have better efficacy and survival regardless of T790M mutation status.

The mPFS of patients with TP53 wt plus EGFR 19del, TP53 wt plus EGFR 21 L858R, TP53 mut plus EGFR 19del, and TP53 mut plus EGFR 21L858R were 17.2, 12.6, 10.4, and 9.0 months, respectively (Figure S3C). Survival analysis for patients grouped by TP53 and EGFR muts indicated that patients with TP53 mut plus EGFR 21L858R had the worst mOS (11.5 months) compared with patients with TP53 wt plus EGFR 19del (29.3 months), TP53 wt plus EGFR 21L858R (28.0 months), and TP53 mut plus EGFR 19del (24.1 months), respectively (*p* < 0.0001; Figure S3D).

## 3 DISCUSSION

In this study, we retrospectively explored two independent cohorts of NSCLC patients treated with third‐generation EGFR‐TKIs to figure out the impact of TP53 comutation. We found that TP53 mutation was related to worse clinical outcomes. Interestingly, TP53 mut in the α‐helix region was associated with poor OS compared with other TP53 mutations. As far as we know, this is the first study to explore the effect of TP53 mut in α‐helix region on clinical outcomes.

In SYSUCC and GENIE cohorts, 50.4−63.8% of patients harbored TP53 comutation, similar to previous studies.^15^, ^17^ We discovered that TP53 mut was related to inferior OS and PFS in patients treated with third‐generation EGFR‐TKIs, which is consistent with reported studies.^15^, ^17^ TP53 mutation was also reported to affect the response and survival of ALK‐ or ROS1‐TKIs treatment.^26^, ^27^, ^28^ TP53 mutations that occur with different patterns and/or distinct comutations could produce interesting functional and phenotypic ramifications,^29^, ^30^ while the impact of different structural TP53 mutations on prognosis remains unclear. Recently, there has been a succession of studies exploring the prognostic effect of TP53 mutation subclassification, one perspective being different exons.^16^, ^20^ TP53 exon 8 mut was reported to be associated with poor OS of first‐generation EGFR‐TKIs.^16^, ^20^ At present, there are not many studies on the effect of TP53 exon 8 muton clinical outcomes of third‐generation TKI (Table S3).^14^, ^15^, ^16^, ^17^, ^31^, ^32^ Canale et al.^16^ reported that mutations in the TP53 exon 8 are related to significantly worse OS. Roeper et al.^15^ found no difference between TP53 exon 8 mutation and PFS or OS, which may be related to the small sample size. In our SYSUCC cohort, TP53 exon 8 was associated with worse PFS but not OS after third‐generation TKI treatment. The influence of TP53 exon 8 in third‐generation TKIs needs to be further explored. Another perspective focuses on the TP53 protein structure, different structured domains have unique properties and contribute to the functional diversity of p53.^25^ Some studies found that TP53 nondisruptive mutations were related to the efficacy of patients receiving first‐line EGFR‐TKIs treatment,^11^, ^20^, ^33^ while few studies have explored the impact of TP53 disruptive mut on third‐generation TKI treatment.^15^ Roeper et al.^15^ observed that the PFS for the TP53 disruptive mutation was a bit shorter than the nondisruptive mutations group (8 vs. 11 months). In our study, despite the shorter PFS observed, the differences between disruptive mutations and nondisruptive mutations were not statistically different. Further exploration is needed on the utility of TP53 disruptive mut in patients treated with third‐generation TKI.

Understanding structural changes can help identify new therapeutic strategies for people with TP53 comutation. In our study, we first explored the impact of mutations related to the secondary structure of protein on clinical outcomes, including α‐helix, β‐strand, loop, turn and others. We found that TP53 mutations in the α‐helix region had a shorter mOS and verified this finding in another independent cohort. α‐Helix is an important protein secondary structural unit, which is vital for the structure and function of the p53 protein.^34^, ^35^, ^36^ α‐Helix plays a role in DNA binding motifs, such as zinc finger structure, leucine zipper, helical‐angle‐helix, and other motifs.^34^, ^35^, ^36^ The composition and sequence of amino acids in protein molecules have decisive influences on the formation and stability of the α‐helix, thus mutations in amino acid sequences in these regions are more likely to result in the instability and interruption of the helix, which can affect the function of p53.^37^ Our finding that TP53 mutations in the α‐helix region are associated with poor clinical outcomes, suggests that this population is of concern, and the α‐helix region may be a potential therapeutic target. It is worth mentioning that this is only a clinical finding, which needs to be validated by a cohort with larger sample sizes, and further mechanistic exploration is needed.

Some studies have shown that patients with different EGFR mutation genotypes have different responses to EGFR‐TKIs,^38^, ^39^ while the effects of concurrent TP53 mutations on TKI efficacy in patients with different genotypes of mutant EGFR remains unclear. Yu et al.^33^ reported that in patients with TP53 comutations who underwent gefitinib treatment, patients harboring EGFR exon 19 deletion had a longer PFS and OS compared with those with EGFR L585R mutation. In our study, patients with TP53 mut plus EGFR L858R had the worst mOS (11 months), which is consistent with the report of Yu et al.^33^ However, Canale et al.^16^ reported that TKI‐treated patients with EGFR exon 19 deletion had shorter PFS and OS than those without 19 deletion. More studies are needed to figure out the relationship between EGFR subtypes and TP53 comutations on the efficacy of EGFR‐TKI.

This study has limitations that must be addressed. Different sizes of NGS panels were applied since the panels have been developed in the laboratory during the study process. The effect of TP53 comutations on specific EGFR‐TKIs, including avitinib or almonertinib, remains unclear and warrants further comprehensive research. More comprehensive experiments are expected to discover the potential mechanism behind the mutant p53 that exerts on the EGFR‐TKIs.

## 4 CONCLUSION

TP53 comutation was an independent negative predictive factor of poor outcomes in advanced EGFR‐mutant NSCLC patients who underwent third‐generation EGFR‐TKIs treatment. Our analyses in two independent cohorts identified that TP53 mutation in α Helix region was associated with inferior outcomes. It is expected that more future studies will explore the impact of structural and functional differences among specific TP53 mutations on patient outcomes, which might potentially make TP53 comutated EGFR‐mutant NSCLC more actionable and targetable in a better precision‐based era.

## 5 METHODS

### 5.1 Patient characteristics

We retrospectively collected 223 patients diagnosed with stage IV NSCLC who received third‐generation EGFR‐TKI therapy at SYSUCC from March 2, 2016 to April 16, 2019. Patients with available sequencing data were included, who were routinely assessed for targeted genetic alterations using tissue, blood, or pleural effusion samples before treatment.

Patients were required to have baseline computed tomography scanning, at least one follow‐up computed tomography scanning, and follow‐up information. Baseline clinical information (age, gender, histology, metastatic sites, smoking history, details of EGFR mutation, TKI selection, treatment lines, and current survival status), medical and imaging records, and patient follow‐up information were assessed. The protocol and amendments were approved by the Ethical Committee of SYSUCC (GZJZ‐SB2016‐010), and written informed consent was obtained from patients.

### 5.2 EGFR and TP53 mutation screening using NGS

DNA was extracted from 5 mL plasma, 200−300 mL pleural effusion, or 15−20 slides of formalin‐fixed and paraffin‐embedded tissue. The specific DNA extraction Kit is based on the actual use of the company, as detailed in Table S4. DNA sequencing was conducted within three Clinical Laboratory Improvement Amendments‐certified labs (MyGene, Burning Rock, BGI‐Shenzhen) or the central laboratory of SYSUCC using NGS. Genomic alterations included single nucleotide variations, deletions, insertions, copy number variations, and gene rearrangements. The MyGene panels covered at least 22 cancer‐related genes; the Burning Rock panels covered 168 cancer‐related genes; the BGI panels covered 206 or 508 cancer‐related genes; and the central laboratory of SYSUCC panels covered 295 cancer‐related genes (Table S5). The minimum coverage across all samples was ≥1000×.^40^

Disruptive/nondisruptive TP53 mut were classified according to mutations located inside the key DNA‐binding domain (L2–L3 region) as previously described.^8^ We also paid attention to the influence of the other secondary structure of TP53 protein, in which TP53 mut were classified according to α‐helix, β‐strand, loop, and turn and others according to the structural features annotating online (Table S2, http://p53.fr/tp53‐database/mutation‐database and www.uniprot.org/uniprotkb/P04637).^41^, ^42^, ^43^, ^44^

### 5.3 American Association for Cancer Research Project GENIE database

The American Association for Cancer Research Project GENIE registry contains genomic data and clinical data from thousands of cancer patients.^45^ Data from the NSCLC cohort version 2.0_public data were accessed through the GENIE Biopharma Collaborative Public platform (https://www.synapse.org/#!Synapse:syn27056697). Patients with advanced EGFR‐mutant NSCLC who received osimertinib with tumor genomic data available were included in this study.

### 5.4 Response evaluation

Patients were followed up regularly by telephone and periodic hospital review. Baseline computed tomography scanning and at least one follow‐up imaging test after the TKI treatment were conducted for response evaluation. Tumor response was evaluated according to the Response Evaluation Criteria in Solid Tumors criteria (RECIST), version 1.1.28. The last patient follow‐up was carried out in May, 2021. PFS was considered as the time from the start of a third‐generation TKI treatment (osimertinib/avitinib/almonertinib) until documentation of disease progression or death. OS was considered as the time from the start of treatment until death. Response to TKI treatment was analyzed based on *EGFR* and *TP53* mutation status.

### 5.5 Statistical analysis

Descriptive statistics were used for baseline characteristics. Pearson's and Spearman's chi‐squared test or Fisher's exact test was used for comparisons between the two groups. The Kaplan–Meier method model was used to estimate PFS and OS, and the log‐rank test was used to compare PFS and OS. The Cox proportional regression model was used to identify prognostic factors, to calculate the HR and corresponding 95% CI. A two‐tailed level of significance was set at 0.05. All statistical analyses were performed with SPSS STATISTICS (version 24.0; IBM Corporation), and R (version 4.0.5). Plots were generated with GraphPad Prism (version 7.0; GraphPad Software).

## AUTHOR CONTRIBUTIONS

*Conception and design*: Zhonghan Zhang, Jinhui Xue, Feiteng Lu, Li Zhang, and Hongyun Zhao. *Development of methodology*: Zhonghan Zhang, Jinhui Xue, Feiteng Lu, Li Zhang, and Hongyun Zhao. *Acquisition of data*: Zhonghan Zhang, Jinhui Xue, Yunpeng Yang, Wenfeng Fang, Yan Huang, Shen Zhao, Fan Luo, Jiaxin Cao, Kangmei Zeng, Wenjuan Ma, Jianhua Zhan, Feiteng Lu, Li Zhang, and Hongyun Zhao. *Analysis and interpretation of data*: Zhonghan Zhang, Jinhui Xue, and Feiteng Lu. *Writing, review, and revision of the manuscript*: Zhonghan Zhang, Jinhui Xue, Yunpeng Yang, Wenfeng Fang, Yan Huang, Shen Zhao, Fan Luo, Jiaxin Cao, Kangmei Zeng, Wenjuan Ma, Jianhua Zhan, Feiteng Lu, Li Zhang, and Hongyun Zhao. *Study supervision*: Li Zhang and Hongyun Zhao. All authors have read and approved the final manuscript

## CONFLICT OF INTEREST STATEMENT

The authors declare that there is no conflict of interest.

## ETHICS STATEMENT

The protocol and amendments were approved by the Ethical Committee of Sun Yat‐sen University Cancer Center (GZJZ‐SB2016‐010), and written informed consent was obtained from patients for the use of their clinical records.

## Supporting information

Supporting Information

## Data Availability

The data of this study have been uploaded onto the Research Data Deposit public platform (https://www.researchdata.org.cn), with the approval number RDDA2024261572. The datasets used in the study are available in the supplements or from the corresponding author upon reasonable request.

## References

[1] Soria JC , Ohe Y , Vansteenkiste J , et al. Osimertinib in untreated EGFR‐mutated advanced non‐small‐cell lung cancer. N Engl J Med. 2018;378(2):113‐125. doi:10.1056/NEJMoa1713137 29151359

[2] Zhao S , Zhang Z , Zhan J , et al. Utility of comprehensive genomic profiling in directing treatment and improving patient outcomes in advanced non‐small cell lung cancer. BMC Med. 2021;19(1):223. doi:10.1186/s12916-021-02089-z 34592968 PMC8485523

[3] Yu HA , Arcila ME , Rekhtman N , et al. Analysis of tumor specimens at the time of acquired resistance to EGFR‐TKI therapy in 155 patients with EGFR‐mutant lung cancers. Clin Cancer Res. 2013;19(8):2240‐2247. doi:10.1158/1078-0432.CCR-12-2246 23470965 PMC3630270

[4] Mok TS , Wu YL , Ahn MJ , et al. Osimertinib or platinum‐pemetrexed in EGFR T790M‐positive lung cancer. N Engl J Med. 2017;376(7):629‐640. doi:10.1056/NEJMoa1612674 27959700 PMC6762027

[5] Lu S , Dong X , Jian H , et al. AENEAS: a randomized phase III trial of aumolertinib versus gefitinib as first‐line therapy for locally advanced or metastaticnon‐small‐cell lung cancer with EGFR exon 19 deletion or L858R mutations. J Clin Oncol. 2022;40(27):3162‐3171. doi:10.1200/jco.21.02641 35580297 PMC9509093

[6] Zhou Q , Wu L , Hu P , et al. A novel third‐generation EGFR tyrosine kinase inhibitor abivertinib for EGFR T790M‐mutant non‐small cell lung cancer: a multicenter phase I/II study. Clin Cancer Res. 2022;28(6):1127‐1135. doi:10.1158/1078-0432.Ccr-21-2595 34740925 PMC9365372

[7] Wang J , Wang B , Chu H , Yao Y . Intrinsic resistance to EGFR tyrosine kinase inhibitors in advanced non‐small‐cell lung cancer with activating EGFR mutations. OncoTargets Ther. 2016;9:3711‐3726. doi:10.2147/ott.S106399 PMC4922765 27382309

[8] Molina‐Vila MA , Bertran‐Alamillo J , Gasco A , et al. Nondisruptive p53 mutations are associated with shorter survival in patients with advanced non‐small cell lung cancer. Clin Cancer Res. 2014;20(17):4647‐4659. doi:10.1158/1078-0432.CCR-13-2391 24696321

[9] Jin Y , Shi X , Zhao J , et al. Mechanisms of primary resistance to EGFR targeted therapy in advanced lung adenocarcinomas. Lung Cancer. 2018;124:110‐116. doi:10.1016/j.lungcan.2018.07.039 30268447

[10] Liu S , Yu J , Zhang H , Liu J . TP53 co‐mutations in advanced EGFR‐mutated non‐small cell lung cancer: prognosis and therapeutic strategy for cancer therapy. Front Oncol. 2022;12:860563. doi:10.3389/fonc.2022.860563 35444951 PMC9013831

[11] Hou H , Qin K , Liang Y , et al. Concurrent TP53 mutations predict poor outcomes of EGFR‐TKI treatments in Chinese patients with advanced NSCLC. Cancer Manag Res. 2019;11:5665‐5675. doi:10.2147/cmar.S201513 31417310 PMC6594053

[12] Christopoulos P , Kirchner M , Roeper J , et al. Risk stratification of EGFR(+) lung cancer diagnosed with panel‐based next‐generation sequencing. Lung Cancer. 2020;148:105‐112. doi:10.1016/j.lungcan.2020.08.007 32871455

[13] Yu HA , Suzawa K , Jordan E , et al. Concurrent alterations in EGFR‐mutant lung cancers associated with resistance to EGFR kinase inhibitors and characterization of MTOR as a mediator of resistance. Clin Cancer Res. 2018;24(13):3108‐3118. doi:10.1158/1078-0432.Ccr-17-2961 29530932 PMC6420806

[14] Cheng Y , Ma L , Liu Y , et al. Comprehensive characterization and clinical impact of concomitant genomic alterations in EGFR‐mutant NSCLCs treated with EGFR kinase inhibitors. Lung Cancer. 2020;145:63‐70. doi:10.1016/j.lungcan.2020.04.004 32408134

[15] Roeper J , Christopoulos P , Falk M , et al. TP53 co‐mutations as an independent prognostic factor in 2nd and further line therapy‐EGFR mutated non‐small cell lung cancer IV patients treated with osimertinib. Transl Lung Cancer Res. 2022;11(1):4‐13. doi:10.21037/tlcr-21-754 35242623 PMC8825660

[16] Canale M , Petracci E , Delmonte A , et al. Concomitant TP53 mutation confers worse prognosis in EGFR‐mutated non‐small cell lung cancer patients treated with TKIs. J Clin Med. 2020;9(4). doi:10.3390/jcm9041047 PMC7230306 32272775

[17] Kim Y , Lee B , Shim JH , et al. Concurrent genetic alterations predict the progression to target therapy in EGFR‐mutated advanced NSCLC. J Thorac Oncol. 2019;14(2):193‐202. doi:10.1016/j.jtho.2018.10.150 30391576

[18] Song H , Wu J , Tang Y , et al. Diverse rescue potencies of p53 mutations to ATO are predetermined by intrinsic mutational properties. Sci Transl Med. 2023;15(690):eabn9155. doi:10.1126/scitranslmed.abn9155 37018419

[19] Chen S , Wu JL , Liang Y , et al. Arsenic trioxide rescues structural p53 mutations through a cryptic allosteric site. Cancer Cell. 2021;39(2):225‐239. doi:10.1016/j.ccell.2020.11.013 e8 33357454

[20] Canale M , Petracci E , Delmonte A , et al. Impact of TP53 mutations on outcome in EGFR‐mutated patients treated with first‐line tyrosine kinase inhibitors. Clin Cancer Res. 2017;23(9):2195‐2202. doi:10.1158/1078-0432.Ccr-16-0966 27780855

[21] Poeta ML , Manola J , Goldwasser MA , et al. TP53 mutations and survival in squamous‐cell carcinoma of the head and neck. N Engl J Med. 2007;357(25):2552‐2561. doi:10.1056/NEJMoa073770 18094376 PMC2263014

[22] Hassin O , Nataraj NB , Shreberk‐Shaked M , et al. Different hotspot p53 mutants exert distinct phenotypes and predict outcome of colorectal cancer patients. Nat Commun. 2022;13(1):2800. doi:10.1038/s41467-022-30481-7 35589715 PMC9120190

[23] Roeper J , Falk M , Chalaris‐Rißmann A , et al. TP53 co‐mutations in EGFR mutated patients in NSCLC stage IV: a strong predictive factor of ORR, PFS and OS in EGFR mt+ NSCLC. Oncotarget. 2020;11(3):250‐264. doi:10.18632/oncotarget.27430 32076486 PMC6980625

[24] Pauling L , Corey RB , Branson HR . The structure of proteins; two hydrogen‐bonded helical configurations of the polypeptide chain. Proc Nat Acad Sci USA. 1951;37(4):205‐211. doi:10.1073/pnas.37.4.205 14816373 PMC1063337

[25] Wang H , Guo M , Wei H , Chen Y . Targeting p53 pathways: mechanisms, structures, and advances in therapy. Signal Transd Target Ther. 2023;8(1):92. doi:10.1038/s41392-023-01347-1 PMC9977964 36859359

[26] Costa DB . TP53 mutations are predictive and prognostic when co‐occurring with ALK rearrangements in lung cancer. Ann Oncol. 2018;29(10):2028‐2030. doi:10.1093/annonc/mdy339 30265285 PMC6225888

[27] Canale M , Petracci E , Cravero P , et al. Prognosis of ALK‐rearranged non‐small‐cell lung cancer patients carrying TP53 mutations. Transl Oncol. 2022;23:101471. doi:10.1016/j.tranon.2022.101471 35779323 PMC9253903

[28] Frost N , Christopoulos P , Kauffmann‐Guerrero D , et al. Lorlatinib in pretreated ALK‐ or ROS1‐positive lung cancer and impact of TP53 co‐mutations: results from the German early access program. Therap Adv Med Oncol. 2021;13:1758835920980558. doi:10.1177/1758835920980558 33613692 PMC7876585

[29] Kastenhuber ER , Lowe SW . Putting p53 in context. Cell. 2017;170(6):1062‐1078. doi:10.1016/j.cell.2017.08.028 28886379 PMC5743327

[30] Stockhammer P , Grant M , Wurtz A , et al. Co‐occurring alterations in multiple tumor suppressor genes are associated with worse outcomes in patients with EGFR‐mutant lung cancer. J Thorac Oncol. 2023. doi:10.1016/j.jtho.2023.10.001 PMC11364167 37806385

[31] Steendam CMJ , Veerman GDM , Pruis MA , et al. Plasma predictive features in treating EGFR‐mutated non‐small cell lung cancer. Cancers. 2020;12(11). doi:10.3390/cancers12113179 PMC7692448 33138052

[32] Yang GJ , Li J , Xu HY , et al. Osimertinib for Chinese advanced non‐small cell lung cancer patients harboring diverse EGFR exon 20 insertion mutations. Lung Cancer. 2021;152:39‐48. doi:10.1016/j.lungcan.2020.11.027 33341538

[33] Yu R , Bai H , Li T , et al. TP53 mutations in circulating tumor DNA in advanced epidermal growth factor receptor‐mutant lung adenocarcinoma patients treated with gefitinib. Transl Oncol. 2021;14(9):101163. doi:10.1016/j.tranon.2021.101163 34192651 PMC8254117

[34] Murre C , McCaw PS , Vaessin H , et al. Interactions between heterologous helix‐loop‐helix proteins generate complexes that bind specifically to a common DNA sequence. Cell. 1989;58(3):537‐544. doi:10.1016/0092-8674(89)90434-0 2503252

[35] Freemont PS . The RING finger. A novel protein sequence motif related to the zinc finger. Ann NY Acad Sci. 1993;684:174‐192. doi:10.1111/j.1749-6632.1993.tb32280.x 8317827

[36] Landschulz WH , Johnson PF , McKnight SL . The leucine zipper: a hypothetical structure common to a new class of DNA binding proteins. Science. 1988;240(4860):1759‐1764. doi:10.1126/science.3289117 3289117

[37] Pace CN , Scholtz JM . A helix propensity scale based on experimental studies of peptides and proteins. Biophys J. 1998;75(1):422‐427. doi:10.1016/s0006-3495(98)77529-0 9649402 PMC1299714

[38] Zhang Y , Sheng J , Kang S , et al. Patients with exon 19 deletion were associated with longer progression‐free survival compared to those with L858R mutation after first‐line EGFR‐TKIs for advanced non‐small cell lung cancer: a meta‐analysis. PLoS One. 2014;9(9):e107161. doi:10.1371/journal.pone.0107161 25222496 PMC4164616

[39] Hong W , Wu Q , Zhang J , Zhou Y . Prognostic value of EGFR 19‐del and 21‐L858R mutations in patients with non‐small cell lung cancer. Oncol Lett. 2019;18(4):3887‐3895. doi:10.3892/ol.2019.10715 31516600 PMC6732961

[40] Chakravarty D , Gao J , Phillips SM , et al. OncoKB: a precision oncology knowledge base. JCO Precis Oncol. 2017. doi:10.1200/po.17.00011 2017 PMC5586540 28890946

[41] Soussi T , Leroy B , Taschner PE . Recommendations for analyzing and reporting TP53 gene variants in the high‐throughput sequencing era. Hum Mutat. 2014;35(6):766‐778. doi:10.1002/humu.22561 24729566

[42] Leroy B , Anderson M , Soussi T . TP53 mutations in human cancer: database reassessment and prospects for the next decade. Hum Mutat. 2014;35(6):672‐688. doi:10.1002/humu.22552 24665023

[43] Leroy B , Fournier JL , Ishioka C , et al. The TP53 website: an integrative resource centre for the TP53 mutation database and TP53 mutant analysis. Nucleic Acids Res. 2013;41:D962‐969. doi:10.1093/nar/gks1033. Database issue. 23161690 PMC3531172

[44] Leroy B , Ballinger ML , Baran‐Marszak F , et al. Recommended guidelines for validation, quality control, and reporting of TP53 variants in clinical practice. Cancer Res. 2017;77(6):1250‐1260. doi:10.1158/0008-5472.Can-16-2179 28254861 PMC7457206

[45] AACR Project GENIE . Powering precision medicine through an international consortium. Cancer Discov. 2017;7(8):818‐831. doi:10.1158/2159-8290.Cd-17-0151 28572459 PMC5611790

