# Supplementary material for: Influence of TP53 mutation on efficacy and survival in advanced EGFR‐mutant non‐small cell lung cancer patients treated with third‐generation EGFR tyrosine kinase inhibitors
Source: MedComm (2020). 2024 Jun 2;5(6):e586. doi: 10.1002/mco2.586 (PMC11144614; doi:10.1002/mco2.586)
Supplement: Supplementary file 1 — Supporting Information [file MCO2-5-e586-s001.docx]

**Supplementary Materials for**

**Influence of TP53 Mutation on Efficacy and Survival in Advanced** **EGFR-Mutant Non-small Cell Lung Cancer** **Patients Treated with Third-generation EGFR Tyrosine Kinase Inhibitors**

Running title: Influence of TP53 on Third-generation EGFR-TKIs

Zhonghan Zhang^1#^, Jinhui Xue^2#^, Yunpeng Yang^3^, Wenfeng Fang^3^, Yan Huang^3^, Shen Zhao^3^, Fan Luo ^4^, Jiaxin Cao^5^, Kangmei Zeng^3^, Wenjuan Ma^4^, Jianhua Zhan^1^, Feiteng Lu^6^*, Li Zhang^3^*, Hongyun Zhao^2*^

**Institutional addresses**

1 Department of Experimental Research, State Key Laboratory of Oncology in South China, Collaborative Innovation Center for Cancer Medicine, Sun Yat-Sen University Cancer Center, Guangzhou, China

2 Department of Clinical Research, Sun Yat-sen University Cancer Center, State Key Laboratory of Oncology in South China, Collaborative Innovation Center for Cancer Medicine

3 Department of Medical Oncology, Sun Yat-sen University Cancer Center, State Key Laboratory of Oncology in South China, Collaborative Innovation Center for Cancer Medicine

4 Department of Intensive Care Unit, State Key Laboratory of Oncology in South China, Collaborative Innovation Center for Cancer Medicine, Sun Yat-Sen University Cancer Center, Guangzhou, China;

5 Department of Anesthesiology, State Key Laboratory of Oncology in South China, Collaborative Innovation Center for Cancer Medicine, Sun Yat-Sen University Cancer Center, Guangzhou, China

6 Department of Hematology, Oncology and Cancer Immunology, Charité - Universitätsmedizin Berlin, corporate member of Freie Universität Berlin and Humboldt-Universität zu Berlin, Berlin, Germany

**^*^Please address correspondence to:**

Hongyun Zhao, MD

Department of Clinical Research, Sun Yat-sen University Cancer Center, State Key Laboratory of Oncology in South China, Guangdong Key Laboratory of Nasopharyngeal Carcinoma Diagnosis and Therapy. No. 651 East Dongfeng Road, Guangzhou, 510060, China. Tel: +86-020-87343894; E-mail: [zhaohy@sysucc.org.cn](mailto:zhaohy@sysucc.org.cn).

Li Zhang, MD

Department of Medical Oncology, Sun Yat-sen University Cancer Center, State Key Laboratory of Oncology in South China, Guangdong Key Laboratory of Nasopharyngeal Carcinoma Diagnosis and Therapy. No. 651 East Dongfeng Road, Guangzhou, 510060, China.

Tel: +86-020-8734-2288; E-mail: [zhangli@sysucc.org.cn](mailto:zhangli@sysucc.org.cn)

Feiteng Lu

Department of Hematology, Oncology and Cancer Immunology, Charité - Universitätsmedizin Berlin, corporate member of Freie Universität Berlin and Humboldt-Universität zu Berlin, Berlin, Germany

E-mail: [feiteng.lu@charite.de](mailto:feiteng.lu@charite.de)

Context

[Figure S1 Kaplan-Meier curves of TP53 mutation structural subtypes. 4](#_Toc165246204)

[Figure S2 Kaplan-Meier survival analysis according to the secondary structure of TP53 protein.. 5](#_Toc165246205)

[Figure S3 Kaplan-Meier curves of progression-free survival and overall survival of EGFR and TP53 mutations. 6](#_Toc165246206)

[Table S1 List of Patients with TP53 Mutations, With Smoking History and Genetic Alteration Exon Structure in SYSUCC cohort. 7](#_Toc165246207)

[Table S2 Characteristics of Patients with and without TP53 Mutations in the GENIE Cohort. 9](#_Toc165246208)

[Table S3 Study on the clinical efficacy of third-generation EGFR-TKIs.. 10](#_Toc165246209)

[Table S4 DNA extraction Kit information 11](#_Toc165246210)

[Table S5 Genes Covered and Extent of TP53 Coverage on Solid Tissue Panel. 12](#_Toc165246211)

Figure S1.

Kaplan-Meier curves of TP53 mutation structural subtypes


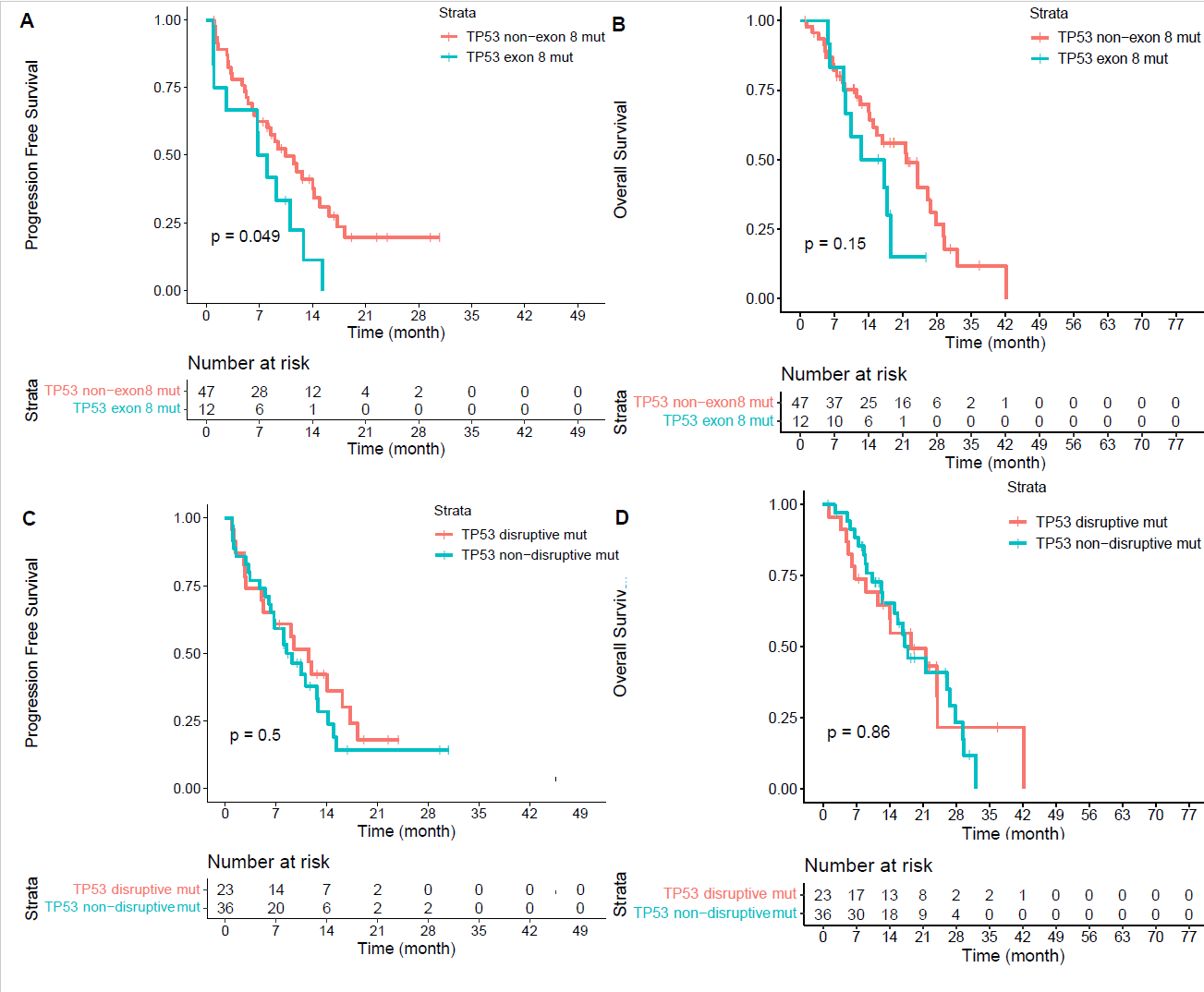


(A) Kaplan-Meier survival analysis of progression-free survival according to the state of TP53 exon 8 mutations. (B) Kaplan-Meier survival analysis of overall survival according to the state of TP53 exon 8 mutations. (C) Kaplan-Meier survival analysis of progression-free survival according to the state of TP53 disruptive mutations. (D) Kaplan-Meier survival analysis of overall survival according to the state of TP53 disruptive mutations.

Figure S2.

Kaplan-Meier survival analysis according to the secondary structure of TP53 protein.


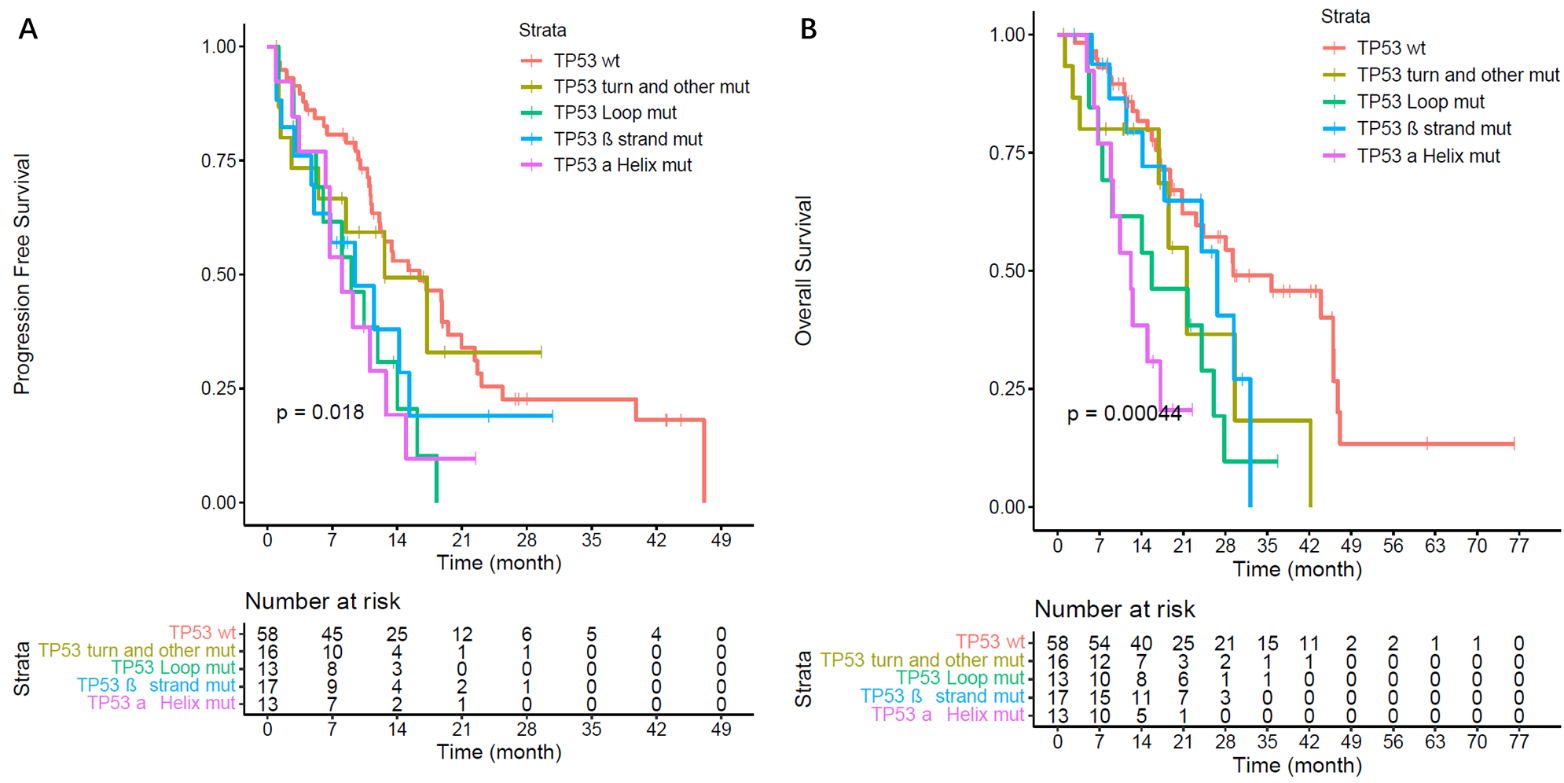


(A) Kaplan-Meier survival analysis of progression-free survival according to the secondary structure of TP53 protein. (B) Kaplan-Meier survival analysis of overall survival according to the secondary structure of TP53 protein.

Figure S3.

Kaplan-Meier curves of progression-free survival and overall survival of EGFR and TP53 mutations


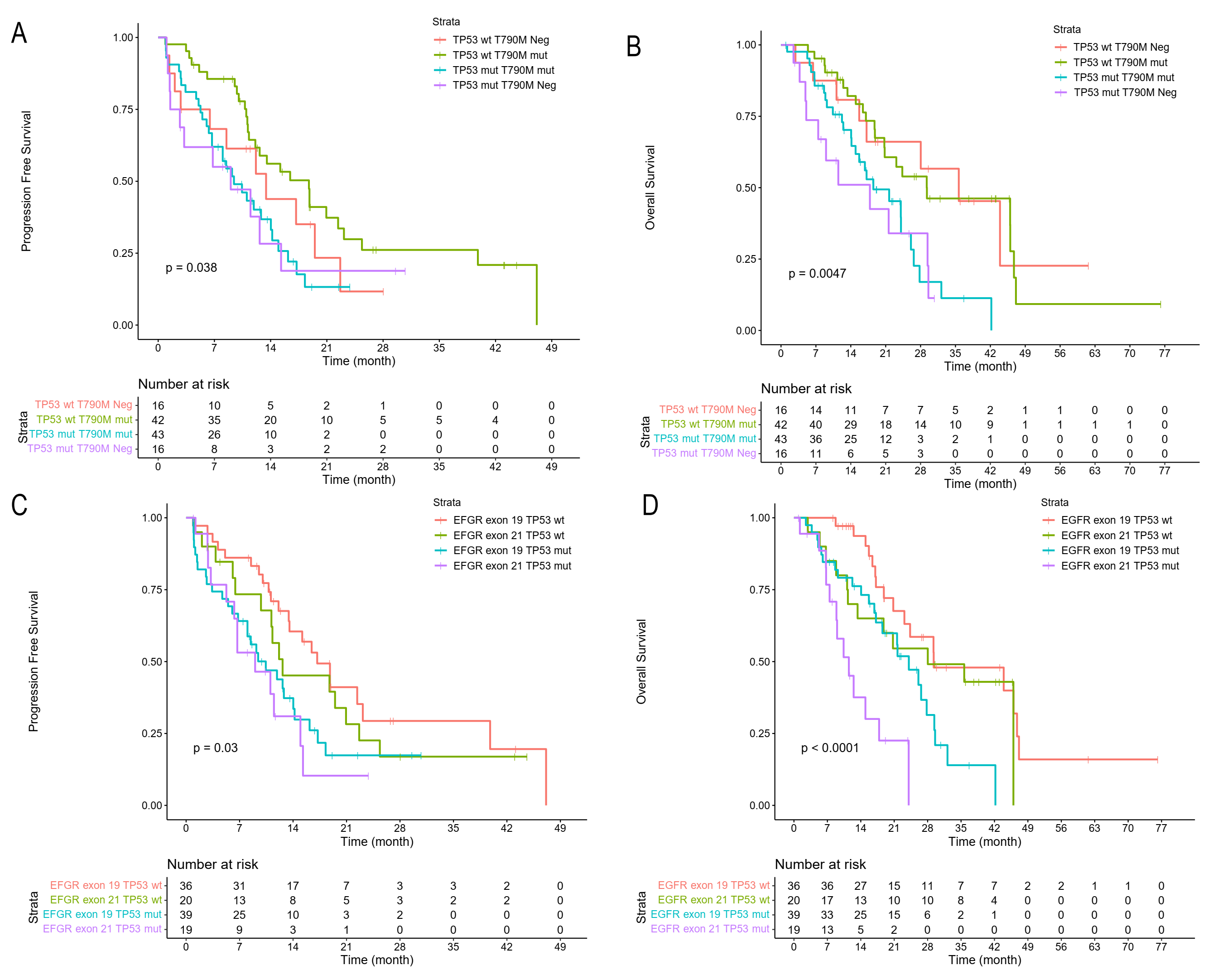


(A) Kaplan-Meier analysis of progression-free survival according to the state of TP53 mutation and T790M mutation. (B) Kaplan-Meier analysis of overall survival according to the state of TP53 mutation and T790M mutation. (C) Kaplan-Meier analysis of progression-free survival analysis according to the state of TP53 mutation and EGFR mutation. (D) Kaplan-Meier analysis of overall survival according to the state of TP53 mutation and EGFR mutation.

Table S1.

List of Patients with TP53 Mutations, With Smoking History and Genetic Alteration Exon Structure in SYSUCC cohort

| Patient ID | Gender | Smoking Status | Mutant p53 Protein | Disruptive subtype | Structure |
| --- | --- | --- | --- | --- | --- |
| 2 | Female | NO | p.D281N | non-Disruptive | α-helix |
| 5 | Male | YES | p.Y220H | non-Disruptive | turn and others |
| 10 | Male | YES | p.Y126S | non-Disruptive | β-strand |
| 15 | Male | NO | p.C176F | Disruptive | loop |
| 18 | Male | YES | p.M237I | non-Disruptive | loop |
| 20 | Female | NO | p.C135Y | non-Disruptive | β-strand |
| 21 | Male | YES | p.Y205C | non-Disruptive | β-strand |
| 28 | Female | NO | p.F270S | non-Disruptive | β-strand |
| 30^*^ | Male | NO | p.R175H  p. D185G | non-Disruptive  Disruptive | loop |
| 32 | Female | NO | p.G244D | Disruptive | loop |
| 34 | Female | NO | p.H179R | non-Disruptive | α-helix |
| 35 | Male | YES | p.P151T | non-Disruptive | turn and others |
| 38 | Female | NO | p.R248L | Disruptive | loop |
| 39 | Female | NO | p.Y220C | non-Disruptive | turn and others |
| 40 | Female | NO | p.R196P | non-Disruptive | β-strand |
| 44 | Female | NO | p.L111P | non-Disruptive | β-strand |
| 48 | Female | NO | p.P151T | non-Disruptive | turn and others |
| 49 | Female | NO | p.R175H | non-Disruptive | loop |
| 50 | Female | NO | p.E285V | non-Disruptive | α-helix |
| 60 | Female | NO | p.Y234Ter | Disruptive | β-strand |
| 64 | Male | YES | p.H214R | non-Disruptive | β-strand |
| 67 | Female | NO | p.D186G | Disruptive | turn and others |
| 69 | Female | NO | p.V272G | non-Disruptive | β-strand |
| 70 | Male | YES | p.Y220C | non-Disruptive | turn and others |
| 71 | Female | NO | p.R248Q | non-Disruptive | loop |
| 74 | Female | NO | p.H179R | non-Disruptive | α-helix |
| 77 | Female | NO | p.E286* | Disruptive | α-helix |
| 78 | Female | NO | p.Q331* | Disruptive | β-strand |
| 79 | Male | YES | p.G245D | Disruptive | loop |
| 81 | Female | NO | p.G245V | Disruptive | loop |
| 84 | Male | NO | p.G266V | non-Disruptive | β-strand |
| 89 | Female | NO | p.S185fs | Disruptive | turn and others |
| 91 | Male | YES | p.K305* | Disruptive | turn and others |
| 95 | Female | NO | p.D228Vfs* | Disruptive | β-strand |
| 96 | Female | NO | p.V272M | non-Disruptive | β-strand |
| 97 | Male | YES | p.Q144* | Disruptive | β-strand |
| 109 | Male | NO | p.M246V | non-Disruptive | loop |
| 110 | Female | NO | p.L130Nfs* | Disruptive | turn and others |
| 118 | Female | NO | p.Y220C | non-Disruptive | turn and others |
| 120 | Male | YES | p.Q192* | Disruptive | turn and others |
| 124 | Male | YES | p.P278A | non-Disruptive | α-helix |
| 125 | Female | NO | p.H179D | Disruptive | α-helix |
| 126 | Male | YES | p.P151A | non-Disruptive | turn and others |
| 128 | Female | NO | p.D259Y | non-Disruptive | turn and others |
| 130 | Male | YES | p.E339fs | Disruptive | α-helix |
| 133 | Male | YES | p.R249Sfs* | Disruptive | loop |
| 135 | Male | YES | p.L137P | non-Disruptive | turn and others |
| 137 | Female | NO | p.I195T | Disruptive | β-strand |
| 138 | Male | YES | c.376-1G>A | Disruptive | turn and others |
| 139 | Female | NO | p.S183* | Disruptive | β-strand |
| 141 | Male | YES | p.R283P | non-disruptive | α-helix |
| 145 | Female | NO | p.N345fs | Disruptive | α-helix |
| 6 | Female | NO | p.L114fs | Disruptive | loop |
| 11 | Male | YES | p.P190L | Non-disruptive | turn and others |
| 25 | Female | NO | p.R280I | Non-disruptive | α-helix |
| 33 | Male | YES | p.F134I | Non-disruptive | β-strand |
| 59 | Female | NO | p.F113V | Non-disruptive | loop |
| 63 | Female | NO | p.L344P | Non-disruptive | α-helix |
| 146 | Female | NO | p.R280G | Non-disruptive | α-helix |

* This patient had both Disruptive and non-disruptive mutations and was included in the disruptive group

Table S2.

Characteristics of Patients with and without TP53 Mutations in the GENIE Cohort

| **No. of patients (%)** | **level** | **Overall** | **TP53 wt** | **TP53 mut in other region** | **TP53 mut in α-helix region** | **P value** |
| --- | --- | --- | --- | --- | --- | --- |
| **Total** |  | 141 | 51 | 73 | 17 |  |
| Race (%) | Asian | 36 (25.5) | 13 (25.5) | 18 (24.7) | 5 (29.4) | 0.481 |
|  | Other | 19 (13.5) | 9 (17.6) | 10 (13.7) | 0 (0.0) |  |
|  | White | 86 (61.0) | 29 (56.9) | 45 (61.6) | 12 (70.6) |  |
| Gender (%) | Female | 99 (70.2) | 39 (76.5) | 47 (64.4) | 13 (76.5) | 0.292 |
|  | Male | 42 (29.8) | 12 (23.5) | 26 (35.6) | 4 (23.5) |  |
| Age (median [IQR]) | | 64.0 [57.0, 74.0] | 65.0 [60.0, 75.0] | 63.0 [56.0, 71.0] | 61.0 [59.0, 68.0] | 0.143 |
| Smoking (%) | No | 74 (52.5) | 26 (51.0) | 36 (49.3) | 12 (70.6) | 0.276 |
|  | Yes | 67 (47.5) | 25 (49.0) | 37 (50.7) | 5 (29.4) |  |
| Histology (%) | Adenocarcinoma | 112 (96.6) | 41 (97.6) | 60 (98.4) | 11 (84.6) | **1** ^a^ |
| **(NA=25)** ^b^ | Squamous cell | 4 (3.4) | 1 (2.4) | 1 (1.6) | 2 (15.4) |  |
| EGFR (%) | **EGFR19** | 80 (56.7) | 26 (51.0) | 43 (58.9) | 11 (64.7) | 0.383 |
|  | **EGFR20** | 14 (9.9) | 7 (13.7) | 5 (6.8) | 2 (11.8) |  |
|  | **EGFR21** | 40 (28.4) | 15 (29.4) | 23 (31.5) | 2 (11.8) |  |
|  | Others | 7 (5.0) | 3 (5.9) | 2 (2.7) | 2 (11.8) |  |
| **Treatment lines (%)** | **First-line** | 13 (9.2) | 3 (5.9) | 9 (12.3) | 1 (5.9) | 0.504 |
|  | **Second-line** | 55 (39.0) | 23 (45.1) | 24 (32.9) | 8 (47.1) |  |
|  | **≥ Third-line** | 73 (51.8) | 25 (49.0) | 40 (54.8) | 8 (47.1) |  |
| **Metastatic sites** |  |  |  |  |  |  |
| **Brain (%)** | No | 61 (63.5) | 20 (64.5) | 34 (69.4) | 7 (43.8) | 0.179 |
| **(NA=45)** | Yes | 35 (36.5) | 11 (35.5) | 15 (30.6) | 9 (56.2) |  |
| **Liver (%)** | No | 78 (81.2) | 24 (77.4) | 44 (89.8) | 10 (62.5) | 0.701 ^a^ |
| **(NA=45)** | Yes | 18 (18.8) | 7 (22.6) | 5 (10.2) | 6 (37.5) |  |
| **Bone (%)** | No | 44 (45.8) | 18 (58.1) | 23 (46.9) | 3 (18.8) | 0.149 ^a^ |
| **(NA=45)** | Yes | 52 (54.2) | 13 (41.9) | 26 (53.1) | 13 (81.2) |  |
| **Pleural effusion (%)** | No | 62 (64.6) | 17 (54.8) | 32 (65.3) | 13 (81.2) | 0.198 |
| **(NA=45)** | Yes | 34 (35.4) | 14 (45.2) | 17 (34.7) | 3 (18.8) |  |
|  |  |  |  |  |  |  |

^a^ Chi-square tests for TP53mut versus TP53wt cases. ^b^ NA,

Abbreviation: IQR, interquartile range; EGFR, epidermal growth factor receptor; TP53 wt, TP53 wild-type; TP53 mut, TP53 mutation; NA, not available

Table S3.

Study on the clinical efficacy of third-generation EGFR-TKIs.

| First author, year | Country | Type of study | Histological type | Patients used third-generation EGFR-TKIs | Line | TP53 and outcome | EGFR mutation profile and outcome |
| --- | --- | --- | --- | --- | --- | --- | --- |
| Kim, 2019 | **Korea** | Retro | Adenocarcinoma (98%) | Osimertinib (n=43) | ≥2nd | TP53 mut vs wt | EGFR 19 del vs L858R |
|  |  |  |  | Olmutinib (n=39) |  | PFS 8.9m vs 12.8m, p=0.029 |  |
|  |  |  |  | TP53（n = 50） |  | OS 17.8m vs 26.6m, p=0.007 |  |
| Roeper, 2022 | **Germany** | Retro | Adenocarcinoma (99%) | Osimertinib（n=77） | ≥2^nd^ | TP53 mut vs wt | EGFR 19 del vs L858R |
|  |  |  |  | TP53（n=32） |  | PFS 9 m vs 14 m, p=0.008 | PFS 10 m vs 11 m, p=0.957 |
|  |  |  |  |  |  | OS 16m vs 24m, p=0.025 | OS 22m vs 15m, p=0.223 |
|  |  |  |  |  |  | TP53 exon 8 mut, Non-exon 8 mut vs wt |  |
|  |  |  |  |  |  | PFS 10m, 9m vs 14m, p=0.017 |  |
|  |  |  |  |  |  | OS 27m,15m vs 24m p=0.081 |  |
|  |  |  |  |  |  | TP53 disruptive mut, non-disruptive mt vs wt |  |
|  |  |  |  |  |  | PFS 8m, 11m vs 14m p=0.011 |  |
|  |  |  |  |  |  | OS 16m, 15m vs 24m p=0.081 |  |
| Canale, 2020 | **Italy** | Retro | NSCLC | Osimertinib (n=41) | ≥2^nd^ | TP53 exon 8 mut, Non-exon 8 mut vs wt |  |
|  |  |  |  | TP53（n=10） |  | PFS 2.83 m, 16.79 m vs 15.28 m, p=0.304 |  |
|  |  |  |  |  |  | OS 18.53m, 42.15 m vs 59.92m, p=0.044 |  |
|  |  |  |  |  |  |  |  |
| Cheng, 2020 | **China** | Retro | Adenocarcinoma (99%) | Osimertinib （n=4） | 1st | None | EGFR 19 del vs L858R: |
|  |  |  |  | Avitinib（n=14） |  |  | PFS 6.6 m vs. 5.7 m, p = 0.26 |
|  |  |  |  |  |  |  | OS 26.3 m vs. 10.8 m, p = 0.01 |
| Steendam, 2020 | **The Netherlands** | Pro | NSCLC | Osimertinib（n=19） | ≥2nd | TP53 mut vs wt | None |
|  |  |  |  | TP53 (n=13) |  | PFS 8.8 m vs. 18.8 m p = 0.017 |  |
| Yang, 2021 | **China** | Retro | Adenocarcinoma (97%) | Osimertinib | 1^st^/≥2^nd^ | TP53 mut vs wt | All EGFR exon 20 insertion mut |
|  |  |  |  | (n=62) |  | PFS 2.2 m vs. 3.7 m p = 0.017 |  |
|  |  |  |  | TP53(n= 37) |  |  |  |

Abbreviation: EGFR-TKIs, epidermal growth factor receptor tyrosine kinase inhibitors; Retro, retrospective; Pro, prospective; TP53 wt, TP53 wild-type; TP53 mut, TP53 mutation;

Table S4

DNA extraction Kit information

| Sample. NO. /Sample Type | Tissue | Plasma | Pleural effusion | DNA extraction Kit |
| --- | --- | --- | --- | --- |
| BGI panels covered 206 or 508 lung cancer-related genes | 11 | 14 | 6 | MGIEasy FFPE DNA Extraction Prepacked Kit (CAT.No 940-000113-00)  MGIEasy Circulating DNA Isolation Kit (CAT.No 1000017017)  MGIEasy Magnetic Beads Genomic DNA Extraction Kit (CAT. 1000010524) |
| Burning Rock panels covered 168 lung cancer-related genes | 11 | 17 | 8 | Internal kit of Burning Rock (https://us.brbiotech.com/products/) |
| MyGene panels covered 22/24/143 lung cancer-related genes | 34 | 8 | 2 | MagPure FFPE DNA/RNA Kit（CAT.No IVD3026）;  MagPure Fast Blood DNA Kit (CAT.No D631001C);  HiPure Tissue DNA Kit (CAT.No D312102) |
| SYSUCC panels covered 295 cancer-related genes | 4 | 2 | - | Kactus Biosystems, Douvity Automed Liquid Handing System (DEP48);  Kactus Biosystems, One-step extraction kit for genomic DNA from FFPE tissue samples (RC1004) |

Table S5.

Genes Covered and Extent of TP53 Coverage on Solid Tissue Panel

| Gene Coverage Panel Version 1 |
| --- |
| AKT1, ALK, APC, AR, ARIDlA,ATM, ATR, B2M, BARD1, BCL2L11,BCOR, BLM, BRAF, BRCA1, BRCA2, BRINP3, BRIP1, CARD11, CASP8, CBL,CCND1, CCNEl, CD274, CD74, CDH18,CDK4, CDK6, CDKN1A, CDKN1B, CDKN2A, CHEK1, CHEK2, CREBBP, CSMD3, CTNNB1,CYP2D6, DIS3, DNMT3A, DPYD, EGFR, EMSY, EP300, EPHA3, EPHA5, EPHA7, EPHB1, ERBB2, ERBB3, ERBB4, ESR1, FANCA, FANCI, FAT3, FBXW7, FGF19, FGF3, FGF4, FGFR1, FGFR2, FGFR3,FLT1,FLT3,FLT4,GATA2,GATA3,GRIN2A,H3F3C,HGF,HIST1H1C,HIST1H3B,HIST1H3G,HRAS,IDH1,IDH2,IGF2, IKZF1, IL7R, INHBA, JAK1, JAK2, KDM5A, KDM6A, KDR, KEAP1, KIT, KMT2D, KRAS, LRP1B, MAP2K1, MAP3K13,MAX,MCL1, MEN1, MET,MLH1,MRE11,MSH2,MSH6, MTOR, MUTYH, MYC, MYCN, NAV3, NBN, NF1, NFE2L2, NOTCH1, NRAS, NRG1, NTRK1, NTRK2, NTRK3, PAK5, PALB2, PARP1, PDGFRA, PDGFRB, PlK3C2G, PlK3C3, PlK3CA, PlK3CG, PIK3R1, PMS2, POLD1, POLE, POM121L12, PPP2R1A, PRKDC, PTEN, PTPRD, PTPRT, RAD50, RAD51B, RAD51C, RAD51D, RAD54L, RAF1, RARA, RB1, RBM10, RET, RNF43, ROS1, RUNXl,SETD2, SMAD4,SMARCA4, SOX2,SOX9,SPOP,SPTA1,SRC,STAG2,STK11,TBX3, TERT, TGFBR2,TP53, TP63,TRIM58, TRPCS, U2AF1,UGT1A1,VEGFA, VEGFB, VEGFC, VHL,YES1 |
| Gene Coverage Panel Version 2 |
| ABL1 ,ABL2 ,ACVR1B ,ACVR2A ,ACVRL1/ALK1 ,AKT1 ,AKT2 ,AKT3 ,ALK ,ALOX12B , AMER1 ,ANGPT1 ,ANGPT2 ,APC ,APCDD1 ,AR ,ARAF ,ARFRP1 ,ARHGAP35 ,ARID1A ,ARID1B ,ARID2 ,ARID5B ,ASXL1 ,ATM ,ATR ,ATRX ,AURKA ,AURKB ,AXIN1 ,AXIN2 ,AXL ,B2M ,B4GALT3 ,BACH1 ,BAK1 ,BAP1 ,BARD1 ,BCL2 ,BCL2A1 ,BCL2L1 ,BCL2L11 ,BCL2L2 ,BCL6 ,BCOR ,BCORL1 ,BCR ,BLM ,BMPR1A ,BRAF ,BRCA1 ,BRCA2 ,BRIP1 ,BTG1 ,BTK ,C11orf30 ,C17orf39/GID4 ,C1QA ,C1R ,C1S ,CARD11 ,CASP8 ,CBFB ,CBL ,CBLB ,CBR1 ,CCND1 ,CCND2 ,CCND3 ,CCNE1 ,CD79A ,CD79B ,CDC25C ,CDC42 ,CDC73 ,CDH1 ,CDK12 ,CDK2 ,CDK4 ,CDK6 ,CDK8 ,CDKN1A ,CDKN1B ,CDKN2A ,CDKN2B ,CDKN2C ,CDX2 ,CEBPA ,CFLAR ,CHD1 ,CHD2 ,CHD4 ,CHEK1 ,CHEK2 ,CHUK ,CIC ,CREBBP ,CRIPAK ,CRKL ,CRLF2 ,CROT ,CSF1R ,CTCF ,CTLA4 ,CTNNA1 ,CTNNB1 ,CUL4B ,CYLD ,CYP19A1 ,CYP2D6 ,CYP3A5 ,CYP2C8 ,CYP3A ,CYP3A4 ,DAXX ,DDR1 ,DDR2 ,DIS3 ,DNMT1 ,DNMT3A ,DOT1L ,DUSP6 ,EDNRA ,EGFR ,EGR3 ,EIF4A2 ,ELAC2 ,ELF3 ,EML4 ,EP300 ,EPCAM ,EPHA2 ,EPHA3 ,EPHA5 ,EPHB1 ,EPHB2 ,EPHB6 ,EPPK1 ,ERBB2 ,ERBB3 ,ERBB4 ,ERCC1 ,ERCC2 ,ERCC3 ,ERG ,ESR1 ,ETV1 ,ETV6 ,EWSR1 ,EXT1 ,EXT2 ,EZH2 ,FAM46C ,FANCA ,FANCC ,FANCD2 ,FANCE ,FANCF ,FANCG ,FANCI ,FANCL ,FANCM ,FAT3 ,FBXW7 ,FCGR1A ,FCGR2A ,FCGR2B ,FCGR3A ,FCGR3B ,FGF10 ,FGF12 ,FGF14 ,FGF19 ,FGF23 ,FGF3 ,FGF4 ,FGF6 ,FGF7 ,FGFR1 ,FGFR2 ,FGFR3 ,FGFR4 ,FH ,FLCN ,FLT1 ,FLT3 ,FLT4 ,FNTA ,FOXA1 ,FOXA2 ,FOXL2 ,FPGS ,FUBP1 , FYN , GAB2 ,GATA1 , GATA2 , GATA3 , GNA11 , GNA13 , GNAQ , GNAS ,GNRHR ,GPR124 ,GRIN2A , GRM3 , H3F3A ,H3F3C , HCK , HDAC1 , HDAC2 , HDAC3 , HDAC4 , HDAC6 ,HDAC8 ,HGF ,HIF1A ,HIST1H1C,HIST1H2BD,HIST1H3B, HNF1A, HRAS, HRH2, HSD17B3, HSD3B2 ,HSP90AA1 , HSPA4, IDH1,IDH2, IFNAR1, IFNAR2, IGF1, IGF1R, IGF2, IKBKB, IKBKE, IKZF1, IL7R, INHBA, IRF4, IRS2, ITGB2, JAK1, JAK2, JAK3, JUB, JUN, KAT6A, KDM5A, KDM5C, KDM6A, KDR, KEAP1, KIAA1549, KIF1B, KIF5B, KIT, KLF4, KLHL6, KMT2A, KMT2B, KMT2C, KMT2D, KNG1, KRAS, KLC3, LCK, LIMK1, LRRK2, LYN, MAP2K1, MAP2K2, MAP2K4, MAP3K1, MAP3K13, MAPK1, MAPK3, MAPK8, MAPK8IP1, MC1R, MCL1, MDM2, MDM4, MECOM, MED12, MEF2B, MEN1, MET, MITF, MLH1, MLH3, MPL, MRE11A, MS4A1, MSH2, MSH3, MSH4, MSH5, MSH6, MSR1, MTOR, MUC1, MUTYH, MYC, MYCL1/MYCL, MYCN, MYD88, MAX, NAV3, NBN, NCOA1, NCOA2, NCOR1, NEK11, NF1, NF2, NFE2L2, NFE2L3, NFKBIA, NOTCH1, NOTCH2, NOTCH3, NOTCH4, NPM1, NR3C1, NRAS, NSD1, NTRK1,NTRK2, NTRK3, NUP93, NKX2-1, NKX3-1, OTOS, PAK3, PAK7, PALB2, PARP1, PARP2, PARP3, PARP4, PAX5, PAX8, PBRM1, PCBP1, PCM1, PD1/PDCD1, PDGFRA, PDGFRB, PDK1, PDL1/CD274, PHF6, PIGF, PIK3C2A, PIK3C2B, PIK3C2G, PIK3C3, PIK3CA, PIK3CB, PIK3CG, PIK3R1,PLK1, PML, PMS1, PMS2, POLQ, PPP2R1A, PRDM1, PRKAA1, PRKAR1A, PRKCA, PRKCB, PRKCG, PRSS8, PSMB1, PSMB2, PSMB5, PTCH1, PTCH2, PTEN, PTP4A3, PTPN11, PTPRD, RAC2, RAD21, RAD50, RAD51, RAD51B, RAD51C, RAD51D, RAD52, RAF1, RARA, RARB, RARG, RB1, REL, RET, RFC1, RHEB, RICTOR, RNASEL, RNF43, ROBO1, ROBO2, ROS1, RPL22, RPL5, RPS14, RPS6KB1, RPTOR, RUNX1, RUNX1T1, RXRA, RXRB, RXRG, SDHAF2, SDHB, SDHC, SDHD, SEMA3A, SEMA3E, SETBP1, SETD2, SF1, SF3B1, SH2B3, SIN3A, SLAMF7, SLC4A1, SLIT2, SMAD2, SMAD3, SMAD4, SMARCA1, SMARCA4, SMARCB1, SMARCD1, SMC1A, SMC3, SMO, SOCS1, SOX10, SOX17, SOX2, SOX9, SPEN, SPOP, SPRY4, SRC, SRD5A2, SRSF2, SSTR2, SSTR5, STAG2, STAT3, STAT4, STAT5B, STK11, SUFU, SUZ12, SYK, TAF1, TBL1XR1, TBX3, TEK, TERT, TET2, TFG, TGFBR2, TIPARP, TMEM127, TNFAIP3, TNFRSF14, TNFRSF8, TNFSF11/RANKL, TNSF13B, TOP1, TOP2A, TOP2B,TP53,TRAF7,TSC1,TSC2,TSHR,TSHZ2,TSHZ3,TUBB,TUBD1,TUBE1,TUBG1,TYR,TLR4,TUBA1A, U2AF1, UMPS, VEGFA, VEGFB, VEZF1, VHL, WHSC1L1, WISP3, WT1, WWP1, XIAP, XPA, XPC, XPO1, XRCC3,YES1, ZNF217, ZNF703, ZRSR2 |
| Gene Coverage Panel Version 3 |
| ABL1,AKT1,AKT2,AKT3,ALK,ALOX12B,AMER1,APC,APCDD1,AR,ARAF,ARFRP1,ARID1A,ARID2,ASXL1,ATM,ATR,ATRX,AURKA,AURKB,AXL,BACH1,BAP1,BARD1,BCL2,BCL2L2,BCL6,BCOR,BCORL1,BCR,BLM,BRAF,BRCA1,BRCA2,BRIP1,BTG1,BT,C110RF30,CARD11,CASP8,CBFB,CBL,CCND1,CCND2,CCND3,CCNE1,CD79A,CD79B,CDC73,CDH1,CDK12,CDK4,CDK6,CDK8,CDKN1B,CDKN2A,CDKN2B,CDKN2C,CEBPA,CHEK1,CHEK2,CHUK,CIC,CRBN,CREBBP,CRKL,CRLF2,CSF1R,CTCF,CTNNA1,CTNNB1,CUL4A,CULAB,CYP17A1,DAXX,DDR2,DIS3,DNMT3A,DOT1L,EGFR,EP300,EPHA3,EPHAS,EPHB1,ERBB2,ERBB3,ERBB4,ERG,ESR1,ETV1,ETV4,ETV5,ETV6,EWSR1,EZH2,FAM46C,FANCA,FANCC,FANCD2,FANCE,FANCF,FANGG,FANCI,FANCL,FANCM,FAT3,FBXW7,FGF10,FGF12,FGF14,FGF19,FGF23,FGF3,FGF4,FGF6,FGF7,FGFR1,FGFR2,FGFR3,FGFR4,FLT1,FLT3,FLT4,FOXL2,GATA1,GATA2,GATA3,GID4,GNA11,GNA13,GNAQ,GNAS,GPR124,GRINZA,GSK3B,HGF,HLA-,HRAS,IDH1,IDH2,IGF1,IGF1R,IGF2,IKBKE,IK2F1,ILTR,INHBA,IRF4,IRS2,JAK1,JAK2,JAK3,JUN,KAT6A,KDM5A,KDM5C,KDM6A,KDR,KEAP1,KIT,KLHL6,KMT2A,KMT2D,KRAS,LMO1,LRP1B,MAP2K1,MAP2K2,MAP2K4,MAP3K1,MAP3K13,MCL1,MDM2,MDM4,MED12,MEF2B,MEN1,MET,MITF,MLH1,MPL,MRE11A,MSH2,MSH6,MTOR,MUTYH,MYC,MYCL1,MYCN,MYD88,NBN,NCOR1,NF1,NF2,NFE2L2,NFKBIA,NKX2-1, NOTCH1, NOTCH2, NOTCH3, NOTCH4, NPM1,NRAS,NSD1,NTRK1,NTRK2,NTRK3,NUP93,PAK3,PAK7,PALB2,PARP1,PARP2,PARP3,PARP4,PAX5,PBRM1,PDGFRA,PDGFRB,PDK1,PIK3C2G,PIK3C3,PIK3CA,PIK3CG,PIK3R1,PIK3R2,PMS2,PNRC1,PPP2R1A,PRDM1,PRKAR1A,PRKDC,PRSS8,PTCH1,PTEN,PTPN11,RAD50,RAD51,RAD51B,RAD510,RAD51D,RAD52,RAD54L,RAF1,RARA,RB1,REL,RET,RICTOR,RNF43,RPA1,RPTOR,ROS1,RUNX1,RUNX1T1,SETD2,SF3B1,SH2B3,SMAD2,SMAD4,SMARCA4,SMARCB1,SMARCD1,SMO,SOCS1,SOX10,SOX2,SPEN,SPOP,SRC,STAG2,STAT4,STK11,SUFU,SYK,TBX3,TET2,TGFBR2,TIPARP,TMPRSS2,TNFAIP3,TNFRSF14,TOP1,TP53,TRRAP,TSC1,TSC2,TSHR,VHL,WISP3,WT1,XPO1,XRCC3,ZNF217,ZNF703 |
| Gene Coverage Panel Version 4 |
| ABL1, ACVRL1, AKT1, AKT2, AKT3, ALK, APC, AR, ARAF, ASPSCR1, ATF1, ATM, ATP11B, ATR, AURKA, BAP1, BCL2, BCL2L1, BCR, BIRC2, BIRC3, BRAF, BRCA1, BRCA2, BRD4, BTK, C11orf30, CBL, CBR3, CCDC6, CCND1, CCND2, CCNE1, CD44, CD74, CDH1, CDK4, CDK6, CDKN2A, CDKN2B, CHEK2, CREB1, CRTC1, CSF1R, CSNK2A1, CTLA4, CTNNB1, DDR1, DDR2, DNMT3A, EGFR, EML4, EPHA2, EPHA3, ERBB2, ERBB3, ERBB4, ERG, ESR1, EZR, ETV6, EWSR1, EZH2, FBXW7, FCGR2A, FCGR2B, FCGR3A, FGD4, FGFR1, FGFR2, FGFR3, FGFR4, FLCN, FLI1, FLT1, FLT3, FLT4, FOXL2, FUS, GAB2, GATA3, GNA11, GNAQ, GNAS, HDAC1, HDAC2, HDAC3, HDAC4, HDAC6, HDAC8, HGF, HNF1A, HRAS, IDH1, IDH2, IGF1R, IL6, IRS2, JAK1, JAK2, JAK3, JAZF1, KDR, KIAA1549, KIF5B, KIT, KRAS, MAML2, MAP2K1, MAPK1, MAX, MCL1, MDM2, MDM4, MED12, MET, MLH1, MLH3, MPL, MS4A1, MSH2, MSH3, MSH6, MTOR, MYB, MYC, MYCN, MYD88, NCOA4, NDRG1, NF1, NF2, NFIB , NOTCH1, NOTCH2, NOTCH3, NOTCH4, NR4A3, NRAS, NTRK3, NUTM1, PARP1, PARP2, PAX5, PAX8, PBX1, PD-1, PDGFRA, PDGFRB, PD-L1, PIK3CA, PIK3CB, PIK3R1, PLAG1, PMS1, POU5F1, PPARG, PPP2R1A, PRCC, PRKAA1, PSMB5, PTCH1, PTEN, PTPN11, RAC1, RAF1, RANKL, RB1, RET, RHEB, RHOA, RICTOR, ROS1, RPS6KB1, SF3B1, SLC34A2, SLC45A3, SMAD2, SMAD4, SMARCA4, SMARCB1, SMO, SND1, SOX2, SPOP, SRC, STAT3, STK11, SUZ12, , TAF15, TCF3, TERT, TET2, TFE3, TMPRSS2, TP53, TPM3, TRIM33, TSC1, TSC2, U2AF1, VEGFA, VHL, WT1, XPO1, ZNF217 |
| Gene Coverage Panel Version 5 |
| ABL1, AKT1, ALK, AR, ARAF, BRAF, BTK, CBL, CDK4, CHEK2, CSF1R,CTNNB1, DDR2, DNMT3A, EGFR, ERBB2, ERBB3, ERBB4, ESR1, EZH2,FGFR1, FGFR2, FGFR3, FLT3, FOXL2, GATA2, GNA11, GNAQ, GNAS,HNF1A, HRAS, IDH1, IDH2, IFITM1, IFITM3, JAK1, JAK2, JAK3, KDR, KIT,KNSTRN, KRAS, MAGOH, MAP2K1, MAP2K2, MAPK1, MAX, MED12,MET, MLH1, MPL, MTOR, MYD88, NFE2L2, NPM1, NRAS, PAX5,PDGFRA, PIK3CA, PPP2R1A, PTPN11, RAC1, RAF1, RET, RHEB, RHOA,SF3B1, SMO, SPOP, SRC, STAT3, U2AF1, XPO1, APC, ATM, BAP1, BRCA1, BRCA2, CDH1, CDKN2A, FBXW7, GATA3,MSH2, NF1, NF2, NOTCH1, PIK3R1, PTCH1, PTEN, RB1, SMAD4,SMARCB1, STK11, TET2, TP53, TSC1, TSC2, VHL, WT1, ACVRL1 , APEX1, AR, ATP11B, BCL2L1, BCL9, BIRC2, BIRC3,CCND1, CCNE1, CD274(PDL1), CD44, CDK4, CDK6, CSNK2A1,DCUN1D1, FGFR4, FLT3, GAS6,IGF1R, IL6, KIT, MCL1, MDM2, MDM4, MYC, MYCL, MYCN,MYO18A, NKX2-1, NKX2-8, PDCD1LG2, PDGFRA, PNP, PPARG,RPS6KB1, SOX2, TERT, TIAF1, ZNF217 |
| Gene Coverage Panel Version 6 |
| AKT1, ALK, BRAF, CTNNB1, DDR2, EGFR, ERBB2(HER2), ERBB4, FBXW7, FGFR1, FGFR2, FGFR3, KRAS, MAP2K1, MET, NOTCH1, NRAS, PIK3CA, PTEN, RET, ROS1, SMAD4, STK11, TP53 |
| Gene Coverage Panel Version 7 |
| AKT1, ALK, BRAF, CTNNB1, DDR2, EGFR, ERBB2(HER2), ERBB4, FBXW7, FGFR1, FGFR2, FGFR3, KRAS, MAP2K1, MET, NOTCH1, NRAS, PIK3CA, PTEN, SMAD4, STK11, TP53 |
